# Rational Construction of Layered Two-Dimensional Conjugated Metal–Organic Frameworks with Room-Temperature Quantum Coherence

**DOI:** 10.1021/jacs.4c18681

**Published:** 2025-02-27

**Authors:** Yang Lu, Yubin Fu, Ziqi Hu, Shiyi Feng, Morteza Torabi, Lei Gao, Shuai Fu, Zhiyong Wang, Chuanhui Huang, Xing Huang, Mingchao Wang, Noel Israel, Evgenia Dmitrieva, Hai I. Wang, Mischa Bonn, Paolo Samorì, Renhao Dong, Eugenio Coronado, Xinliang Feng

**Affiliations:** † Université de Strasbourg, CNRS, ISIS, UMR 7006, 8 Alleé Gaspard Monge, 67000 Strasbourg, France; ‡ Max Planck Institute of Microstructure Physics, 06120 Halle (Saale), Germany; § Center for Advancing Electronics Dresden & Faculty of Chemistry and Food Chemistry, Technische Universität Dresden, 01067 Dresden, Germany; ∥ Instituto de Ciencia Molecular (ICMol), Universitat de València, 46980 Paterna, Spain; ⊥ Department of Materials Science and Engineering, CAS Key Laboratory of Materials for Energy Conversion, Anhui Laboratory of Advanced Photon Science and Technology, University of Science and Technology of China, 230026 Hefei, China; # Max Planck Institute for Polymer Research, 55128 Mainz, Germany; ∇ Leibniz Institute for Solid State and Materials Research, 01069 Dresden, Germany; ○ Nanophotonics, Debye Institute for Nanomaterials Science, Utrecht University, Princetonplein 1, 3584 CC Utrecht, The Netherlands; ◆ Department of Chemistry, The University of Hong Kong, Hong Kong 999077, China; ¶ Materials Innovation Institute for Life Sciences and Energy (MILES), HKU-SIRI, Shenzhen 518048, China; ¶¶ MOE Key Laboratory of Low-grade Energy Utilization Technologies and Systems, School of Energy & Power Engineering, Chongqing University, 400044 Chongqing, China

## Abstract

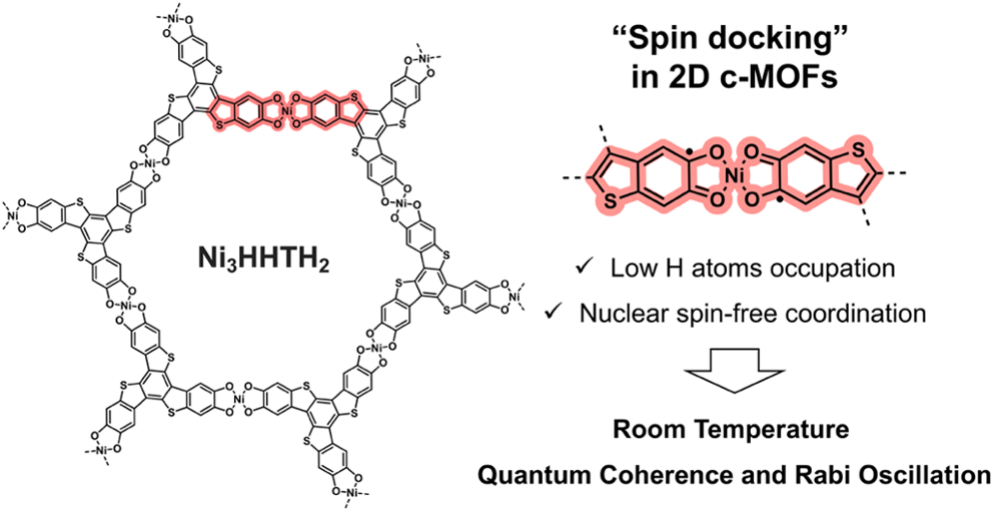

Two-dimensional conjugated metal–organic frameworks (2D c-MOFs) have emerged as an intriguing class of quantum materials due to their high crystallinity, persistent spin centers, and tunable structures and topologies. However, it remains unclear how to achieve long spin relaxation time at room temperature in 2D c-MOFs via a bottom-up design strategy. Herein, we design a hexahydroxytrithiatruxene ligand (HHTH) to minimize the influence of nuclear spin on electron spin relaxation while weakening *d*–π conjugation to construct a “spin docking” for preserving spin centers, which enables the resulting 2D c-MOFs, Ni_3_HHTH_2_, to exhibit quantum coherence and Rabi oscillations at room temperature. Spin dynamics studies not only reveal an unusual temperature-dependent Rabi frequency in Ni_3_HHTH_2_ but also indicate that the coordination mode determines the spin–lattice relaxation behavior via spin–phonon coupling. These investigations provide a general guideline for the development of high-performance quantum qubits based on 2D spin arrays.

## Introduction

Over the past decade, two-dimensional conjugated metal–organic frameworks (2D c-MOFs) have emerged as a new type of *d*-π conjugated van der Waals layered materials, attracting increasing attention due to their unique combination of porosity and electrical conductivity.^1−9^ Significantly, the redox coordination polymerization reaction between π–conjugated ligands featuring adjacent substituent atoms (N, O, and S) and metal ions lead to the creation of persistent radicals on these ligands, endowing 2D c-MOFs with a distinct identity as “spin-concentrated assemblies.”^10−12^ Compared to organic/inorganic molecular spin materials, spin centers in 2D c-MOFs not only exhibit excellent air stability, weak spin–orbit coupling, and hyperfine interactions, but also, more importantly, allow for scalable tuning of spin dynamics.^13−15^ These preliminary explorations position 2D c-MOFs as promising candidates for quantum materials.^10,16^ However, the strong interlayer coupling, combined with intralayer *d*–π conjugation, accelerates spin relaxation across the entire framework.^17^ The consequent low spin concentration (the number of unpaired electron spins per unit of the material) and rapid spin relaxation severely limit the potential of 2D c-MOFs as robust spin qubit arrays.

The dynamics of molecular spins are predominantly governed by their surrounding chemical environment, characterized by two key processes: (i) energy transfer between the spin and its environment (e.g., the ’lattice’), quantified by the spin–lattice relaxation time (*T*_1_); and (ii) spin decoherence, described by the quantum coherence time (*T*_2_).^14,15,18^ In principle, modulating the *d*-π conjugated structures of 2D c-MOFs, which constitute the local connection sphere of the spin, could significantly influence the spin distribution, thereby controlling the spin relaxation process.^10^ On the other hand, the spin-dephasing process is susceptible to losses induced by nuclei, such as hydrogen or nitrogen atoms in the conjugated framework, through hyperfine interactions between electrons and nuclear spins.^16,19−25^ Up to now, it is still highly challenging to develop molecular design strategies toward long spin–lattice and spin-decoherence times at room temperature in 2D c-MOFs.^13,17^

**Figure 1:**
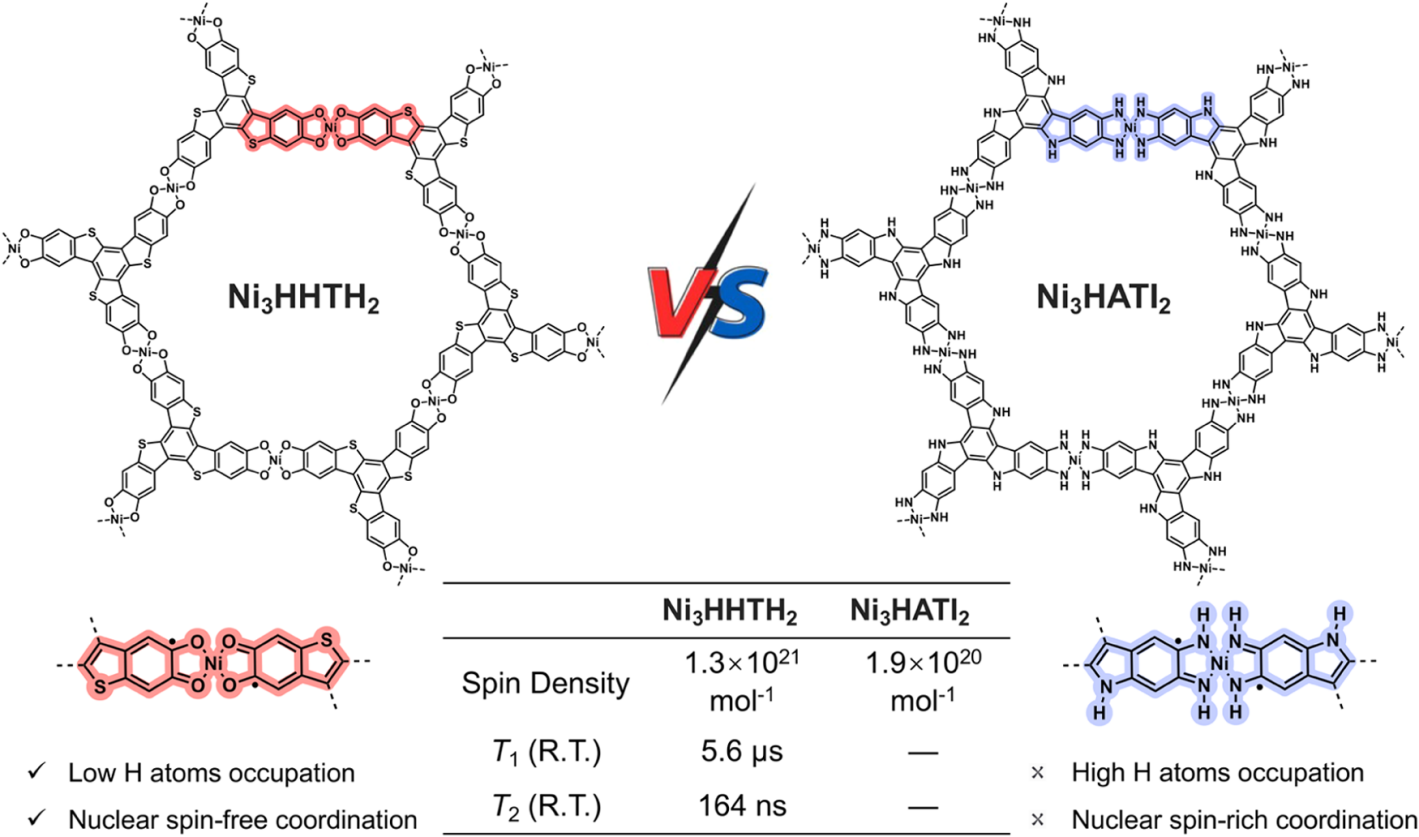
Rational design concept of Ni_3_HHTH_2_ and Ni_3_HATI_2_ 2D c-MOFs (top). Comparation of key static and dynamic spin properties between Ni_3_HHTH_2_ and Ni_3_HATI_2_ 2D c-MOFs (bottom table).

Herein, we report a novel 2D c-MOF based on a π-conjugated ligand, benzo[1,2-*b*:3,4-*b*′:5,6-*b*″]tris[1]benzothiophene-2,3,7,8,12,13-hexol (HHTH), which possesses a similar π-conjugated structure to the previously reported 2,3,7,8,12,13-hexaiminotriindole (HATI) ligand (Figure 1).^13^ Nuclear spin-free Ni^2+^ is utilized to link the HHTH or HATI ligands, resulting in the formation of the corresponding 2D c-MOFs, Ni_3_HHTH_2_ and Ni_3_HATI_2_, respectively, which exhibit identical topology and interlayer stacking modes. Compared to Ni_3_HATI_2_, Ni_3_HHTH_2_ has 60% fewer hydrogen atoms; and nuclear spin-free oxygen and sulfur atoms replace the nitrogen atoms with nuclear spin in Ni_3_HATI_2_. Theoretical calculations indicate that the [NiO_4_] secondary building units (SBUs) of Ni_3_HHTH_2_ can serve as a “spin dock”, effectively localizing the spin center and shielding it from rapid relaxation. Pulse electron paramagnetic resonance (EPR) spectroscopy elucidates the spin dynamics of Ni_3_HHTH_2_, with *T*_1_ and *T*_2_ values of 5.6 μs and 164 ns at 300 K, which are comparable with those of classical MOF-based qubits.^13^ An unusual temperature-dependent Rabi frequency is further revealed, which is attributed to the coupling of electron spins to phonons.

## Results and Discussion

The conjugated ligand, HHTH with *C*_3_ symmetry was synthesized through a modified cyclotrimerization of 5,6-dimethoxybenzo[*b*]thiophen-3(2H)-one and further demethylation reactions (refer to the SI for synthesis details). Ni_3_HHTH_2_ was then constructed under solvothermal conditions by reacting HHTH with Ni(acac)_2_ in a 3: 1 (v/v) mixture of H_2_O and dimethylacetamide (DMAc) in a glass vial at 85 °C for 12 h. The synthesis conditions for Ni_3_HATI_2_ were optimized based on the previous report^26^ (see the SI Section S2). X-ray photoelectron spectroscopy (XPS) analysis of the Ni(2p) regions reveals the presence of Ni(II) in both 2D c-MOFs (Figures S1–S2). Fourier transform infrared (FT-IR) spectroscopy reveals the near disappearance of O–H and N–H stretching vibration bands belonging to the ligand in both Ni_3_HHTH_2_ and Ni_3_HATI_2_ (Figure S3), indicating the deprotonation and subsequent formation of Ni–O and Ni–NH coordination bonds, respectively. Thermogravimetric analysis (TGA) reveals that both 2D c-MOFs start desolvation over 100 °C and exhibit pronounced weight losses above 200 °C due to decomposition (Figure S4). Scanning electron microscopy (SEM, Figure S5) showed rod-like nanocrystals.

**Figure 2:**
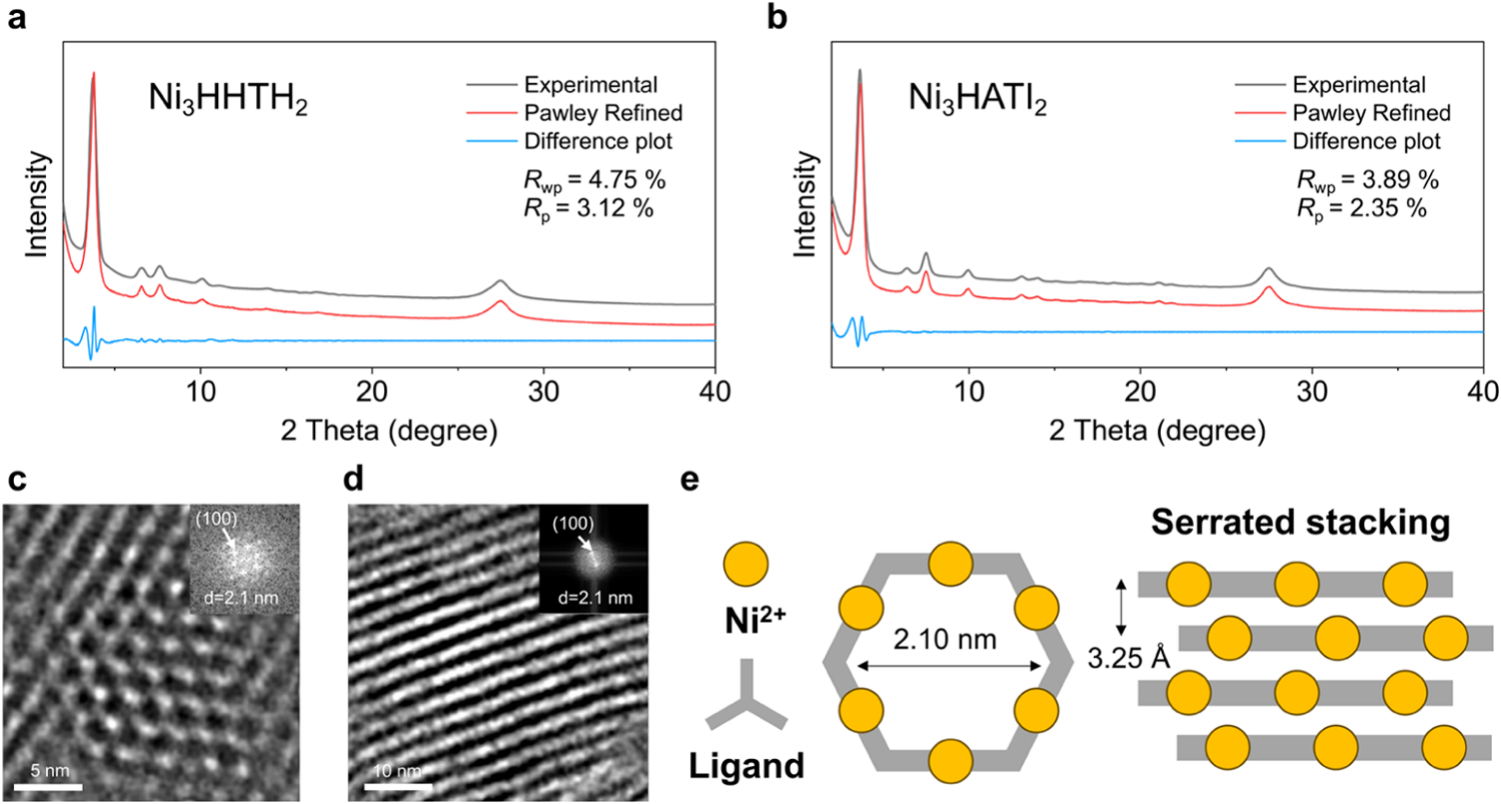
Structural characterization of Ni_3_HHTH_2_ and Ni_3_HATI_2_. (a–b) Pawley refinement of PXRD data with experimental (gray), simulated (red), and difference (blue) patterns for Ni_3_HHTH_2_ and Ni_3_HATI_2_. HRTEM images of Ni_3_HHTH_2_ with fast Fourier transform analysis of the corresponding areas: (c) imaged along the *c* direction and (d) imaged normal to the *c* direction. (e) Schematic stacking mode of Ni_3_HHTH_2_ and Ni_3_HATI_2_.

Powder X-ray diffraction (PXRD) analysis of Ni_3_HHTH_2_ revealed a crystalline phase, evidenced by prominent peaks at 2θ = 3.74, 6.58, 7.57, 10.00, 16.84, and 27.40° (Figure 2a). The PXRD of Ni_3_HATI_2_ (Figure 2b) has a similar pattern to Ni_3_HHTH_2_. The structural elucidation for both samples was achieved through simulation and Pawley refinement of the PXRD experimental data with unit cell parameters listed in Table S1. Comparison of the PXRD pattern and density functional theory (DFT) simulations unveils a long-range growth in the ab plane, accompanied by a serrated layered arrangement characterized by an interlayer shift of 0.1*a and 0.1*b (Figure 2e). The broad peaks centered around 2θ = 27.40° correspond to the (002) plane, revealing an interlayer spacing of ∼3.25 Å in both MOFs. The local structures of the samples were further confirmed by high-resolution transmission electron microscopy (HRTEM). The fast Fourier transform (FFT) analysis of HRTEM images of Ni_3_HHTH_2_ and Ni_3_HATI_2_ presents a honeycomb lattice with a lattice distance of 25.4 Å (Figures 2c–d and S6), which is consistent with the PXRD and calculated results. N_2_ adsorption isotherms at 77 K indicate Brunauer–Emmett–Teller (BET) surface areas of 960 and 1080 m^2^ g^–1^ for Ni_3_HHTH_2_ and Ni_3_HATI_2_, respectively (Figure S7). Both samples possess the average pore size of 2.1 nm obtained by fitting the isotherm, which agrees with the predicted pore structure with diameters of ∼2.1 nm. The achieved results further prove that these samples are isostructural 2D c-MOFs.

**Figure 3:**
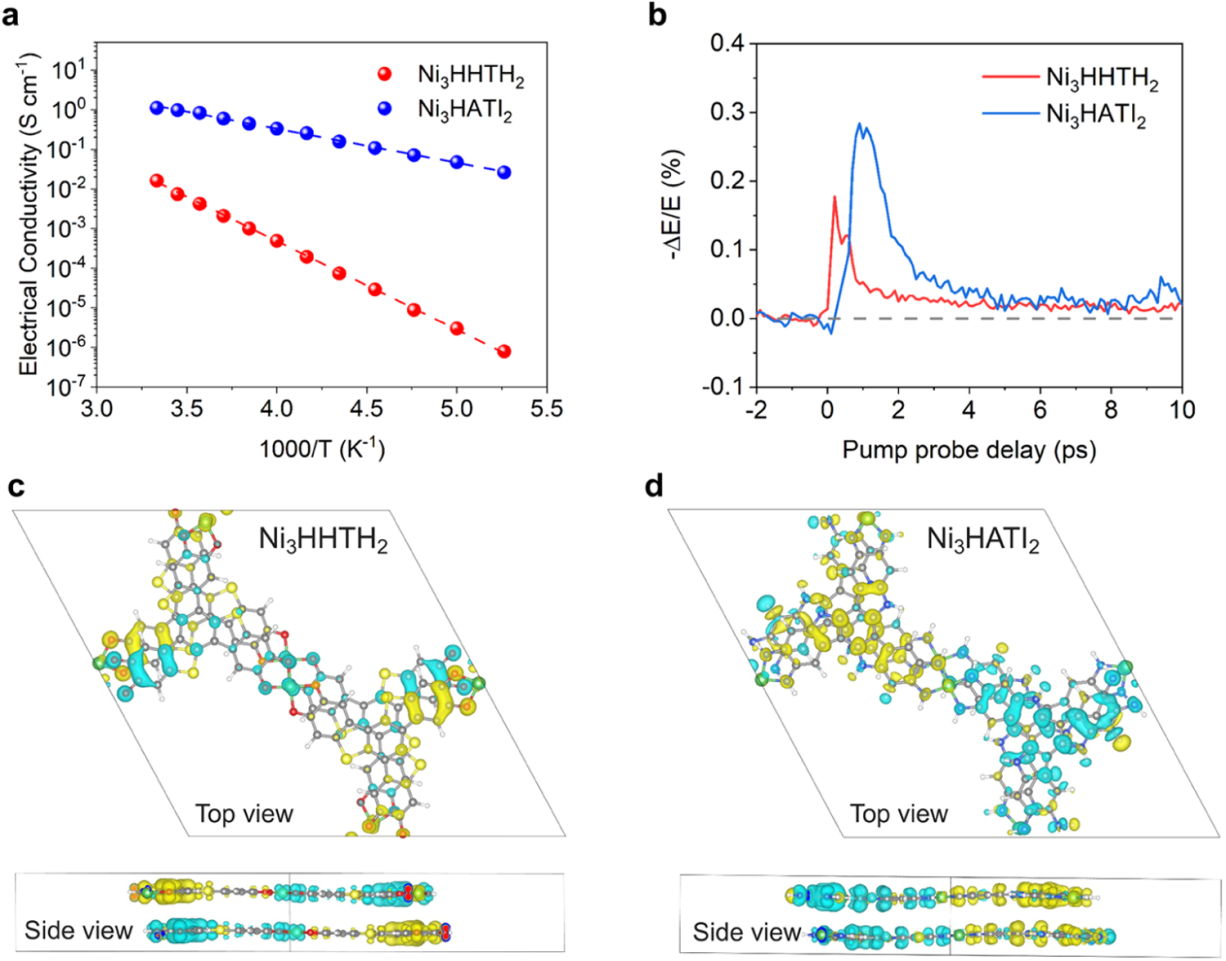
(a) Electrical conductivities of Ni_3_HHTH_2_ and Ni_3_HATI_2_ in the temperature range of 200–320 K. (b) THz photoconductivity dynamics following 3.1 eV photoexcitation (pump fluence: 760 μBC;J/cm^2^). Bulk spin density distributions for (c) Ni_3_HHTH_2_ and (d) Ni_3_HATI_2_, and the yellow and cyan colors denote α and β spin densities, whose iso-value is 0.005 e/Bohr^3^.

Optical absorption reveals that Ni_3_HHTH_2_ exhibits a larger optical bandgap (0.40 eV) than that of Ni_3_HATI_2_ (0.25 eV) (Figure S8). This observation suggests a significant difference in the spin bath of organic radicals generated through coordination of conjugated ligands with Ni ions. The parallel four-probe measurements under ambient conditions showed that the electrical conductivities of Ni_3_HHTH_2_ and Ni_3_HATI_2_ powder pellets at 298 K are 1.6 ± 0.2 × 10^–2^ S cm^–1^ and 1.1 ± 0.1 S cm^–1^, respectively (Figure 3a). Both samples exhibit thermally activated charge transport according to the variable-temperature (VT) conductivity measurements from 200 to 320 K. The activation energy for charge transport in Ni_3_HHTH_2_ is 238 meV, significantly higher than the 45 meV observed in Ni_3_HATI_2_. This substantial disparity further suggests notable differences in charge transport behavior between two isostructural 2D c-MOFs.

To investigate their microscopic transport properties, we employed time-resolved terahertz spectroscopy (TRTS, see SI for details). Ni_3_(HHTH)_2_ and Ni_3_(HATI)_2_ both exhibit a rapid sub-ps rise in photoconductivity due to the quasi-instantaneous injection of free carriers (Figure 3b). This is followed by a ps-level decay, attributed to exciton formation, electron–hole recombination and/or defect trapping.^27,28^ The substantial difference in the photoconductivity amplitude provides preliminary evidence of their distinct charge transport properties. Further analysis on the frequency-resolved complex photoconductivity Δσ(ω) by the phenomenological Drude–Smith (DS) model reveals a substantial difference in the charge scattering time (τ) (∼1–10 fs for Ni_3_HHTH_2_ and 150 ± 10 fs for Ni_3_HATI_2_), which partially explains the photoconductivity discrepancy. The calculated band structures reveal that the band dispersion near the Fermi level along the π-stacking direction in Ni_3_HATI_2_ is significantly larger than in Ni_3_HHTH_2_, indicating that Ni_3_HATI_2_ has considerably stronger interlayer electronic coupling and therefore more efficient carrier transport than Ni_3_HHTH_2_ (Figures S9–S10). The spin distributions obtained from DFT calculations also differ notably between Ni_3_HHTH_2_ and Ni_3_HATI_2_ (Figures 3c–d and S11), with the spin in Ni_3_HATI_2_ being significantly more delocalized across the entire d−π conjugated framework (including the H atoms) compared to Ni_3_HHTH_2_. These results suggest that the extended spin carriers in Ni_3_HATI_2_ interact with the high gyromagnetic ratio H nuclei along transport pathways, simultaneously increasing the likelihood of spin interactions across layers, which may enhance the spin relaxation process. Combined with spin distribution analysis, it is evident that the [NiO_4_] linker in Ni_3_HHTH_2_ acts as a trap in charge transport, resulting in lower transport efficiency. On the other hand, it may also function as a spin docking site, which prevents the spin centers from decoherence. These characteristics can be beneficial for achieving high spin concentration and ultralong spin lifetimes in spin qubit arrays.

**Figure 4:**
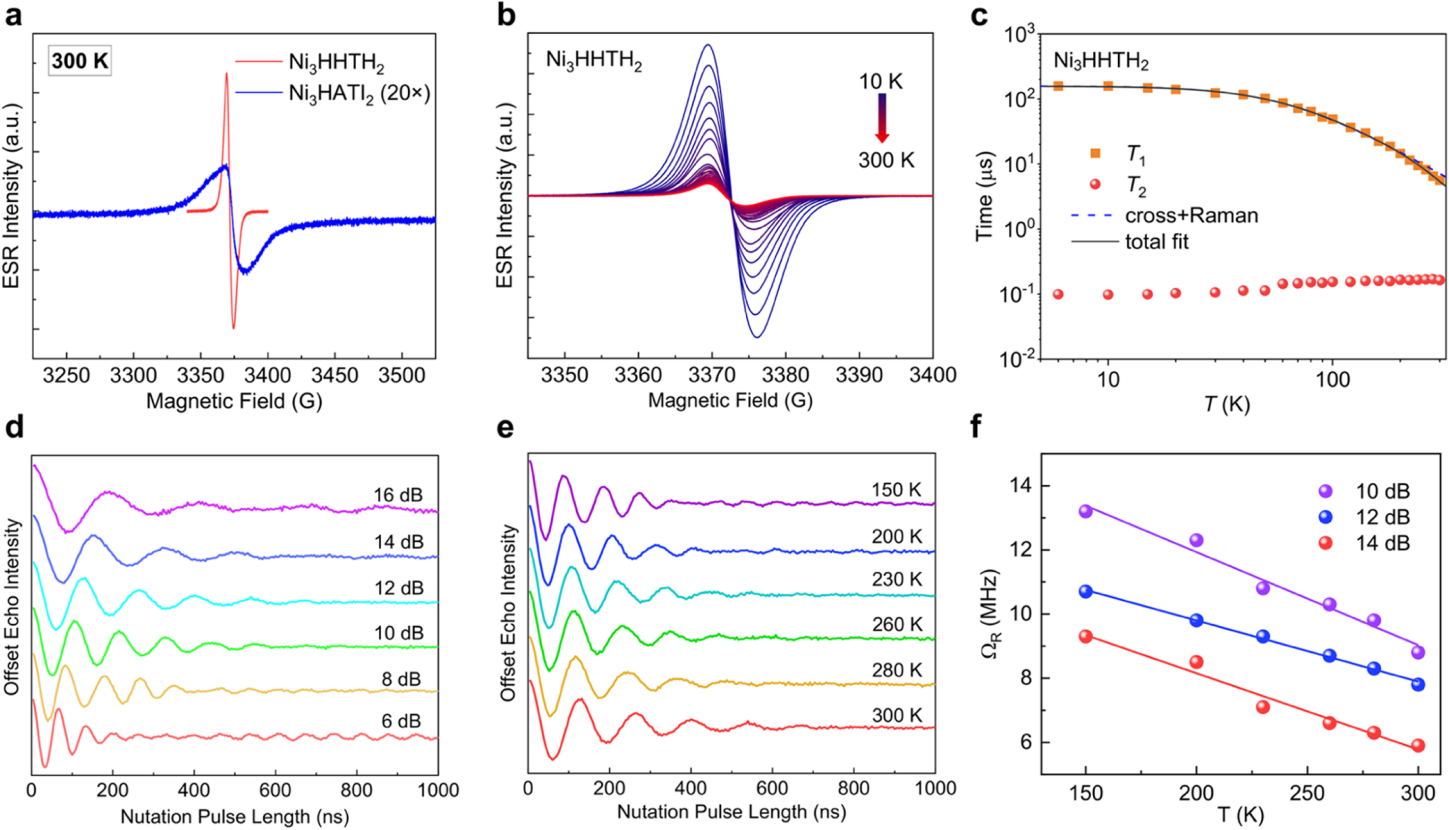
(a) Continuous-wave (CW) EPR spectra of Ni_3_HHTH_2_ and Ni_3_HATI_2_ measured at 300 K. (b) CW-EPR spectra of Ni_3_HHTH_2_ measured at 10–300 K. (c) Temperature dependence of *T*_1_ and *T*_2_ for Ni_3_HHTH_2_ and the fits see text. (d) Normalized echo intensity in nutation experiments using different attenuation powers of the applied microwave at 300 K on Ni_3_HHTH_2_. (e) Nutation experiments on Ni_3_HHTH_2_ at different temperatures using the fixed microwave attenuation power of 12 dB. (f) Temperature dependence of Rabi frequencies determined by nutation experiments at different attenuation powers. The solid lines are guides for the eye.

X-band continuous-wave (CW) EPR spectroscopy was first utilized to probe the spin centers in these 2D c-MOFs. The CW EPR spectrum of Ni_3_HHTH_2_ at room temperature (300 K) displays a single resonance peak at *g* = 2.0063 with a peak-to-peak line width of 5 G. In contrast, a peak at *g* = 2.0052 is identified for Ni_3_HATI_2_ with a much larger line width up to 17 G (Figure 4a). According to the Elliott mechanism, the scattering of high-conduction carriers by phonons in Ni_3_HATI_2_ results in a shortened spin–lattice relaxation time, thereby broadening the EPR signals. This strongly suggests that the spin centers in Ni_3_HHTH_2_ are more localized.^29^ This observation also implies that Ni_3_HHTH_2_ exhibits a longer spin lifetime. Quantitative EPR analysis reveals that Ni_3_HHTH_2_ possesses a spin concentration of 1.3 × 10^21^ mol^–1^, nearly an order of magnitude higher than that of Ni_3_HATI_2_. VT-EPR spectra of the spin-concentrated Ni_3_HHTH_2_ show that the spin signal becomes more intense as temperature decreases (Figure 4b). Doubly integrating the EPR spectra, the temperature dependence of spin susceptibility χ_tot_ in a χ_tot_*T*-*T* plot showcases a clear slope at the temperature region of 50–300 K, revealing significant Pauli contributions from free conducting electrons (Figure S12).

Next, we employed the pulsed EPR technique to characterize the spin dynamics of Ni_3_HHTH_2_ on dry powders. Intriguingly, spin echo signals can be detected at room temperature. Using inversion recovery and Hahn echo decay pulse sequences, *T*_1_ and *T*_2_ were determined to be 5.6 μs and 164 ns at 300 K, respectively, demonstrating long-lived radical centers in a spin-concentrated 2D c-MOF. Such an excellent spin decoherence property is comparable with those of classical MOF-based qubits,^13^ while the vast majority of MOF-based qubits show fragile quantum coherence predominantly at low temperatures.^15,17,30^ In comparison, the spin echo signal for Ni_3_HATI_2_ is too weak to be recorded even at 5 K due to the highly delocalized radicals over [NiNH_4_] and hyperfine coupling with abundant hydrogen atoms in the framework. Upon cooling, the value of *T*_1_ of Ni_3_HHTH_2_ first increases and then becomes nearly temperature-independent below 20 K, reaching up to 158 μs at 6 K (Figure 4c). On the other hand, *T*_2_ remains almost constant and thus is not limited by *T*_1_ in the whole temperature range of 6–300 K since *T*_1_ is sufficiently longer. A similar observation has been reported for molecular graphene nanostructures, which suggests that decoherence likely stems from electron–electron scattering along the π-stacks.^31^ The above-mentioned results point out that the introduction of spin docking sites in 2D c-MOFs, although significantly reducing electrical conductivity from 1.1 S cm^–1^ down to 0.016 S cm^–1^, markedly enhances spin concentration from 1.9 × 10^20^ mol^–1^ to 1.3 × 10^21^ mol^–1^ and achieves room-temperature quantum coherence. This highlights an important design principle for developing 2D c-MOF-based spin qubits.

The temperature dependence of *T*_1_ for Ni_3_HHTH_2_ is fitted by considering cross-relaxation, Raman-like, and local-mode processes using the following expression:
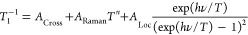
where the first cross-relaxation term is temperature-independent arising from spin–spin interaction,^32^ the second term denotes a power law of the two-phonon Raman mechanism,^33^ and the third term describes the circumstance when a local vibrational mode with a frequency of ν is responsible for spin relaxation via spin–phonon coupling.^34^ Extracted from this analysis, an excellent simulation is obtained with *A*_Cross_ = 6.28 × 10^3^ s^–1^, *A*_Raman_ = 2.62 s^–1^K^–*n*^ and *n* = 1.87 for the Raman-like mechanism, which is attributed to the spin–phonon bottleneck effect,^35^ as well as *A*_Loc_ = 8.81 × 10^5^ s^–1^ and ν = 604 cm^–1^ consistent with the excitation energy (∼606 cm^–1^) of local stretching mode of the Ni–O bonds observed by IR spectroscopy (Figure S13). Note that a combination of cross-relaxation and Raman-like mechanism alone is not able to produce a satisfactory fit in the high-temperature region of 150–300 K (Figure 4c), demonstrating the crucial role of the local vibrational mode in governing spin relaxation of Ni_3_HHTH_2_ at high temperatures.

Nutation experiments of Ni_3_HHTH_2_, showing Rabi oscillation decays (Figure 4d), confirm that arbitrary superposition of spin states can be generated at room temperature with appropriate microwave pulses, establishing Ni_3_HHTH_2_ as an excellent *S* = 1/2 spin qubit candidate for quantum gate operations (Figures S14–S18). This phenomenon is corroborated by the quantitative analysis of spin concentration in Ni_3_HHTH_2_, which reveals that only about 1% of the conjugated ligands contain radicals. This leads to negligible interactions between spin centers, resulting in properties characteristic of nearly isolated free radicals. The Fourier transform of nutation experiments confirms the Rabi oscillations with Rabi frequency Ω_Rabi_ proportional to the magnetic field *B*_1_ of the applied microwave pulse, satisfying the Rabi relationship ℏΩ_Rabi_ = *g*μ_B_*SB*_1_, where ℏ is the reduced Planck constant and μ_B_ is the Bohr magneton. Surprisingly, temperature-dependent Rabi frequency is observed in Ni_3_HHTH_2_ above 150 K, despite the temperature-independent Rabi relationship (Figure 4e–f). This behavior is likely attributed to phonon-induced renormalization of Rabi frequency, a phenomenon previously reported for optically driven semiconducting quantum dots.^36,37^ Such an unprecedented behavior in electron spin qubits suggests strong spin–phonon coupling in this 2D c-MOF, likely driven by the local vibrational mode identified from the temperature dependence of *T*_1_ at similarly high temperatures.

## Conclusions

In summary, we report a novel 2D c-MOF, Ni_3_HHTH_2_ based on a π-conjugated ligand hexahydroxytrithiatruxene ligand by incorporating nuclear spin-free S and O atoms to construct “spin docking” SBU in a 2D *d*-π conjugated system. Ni_3_HHTH_2_ exhibits quantum coherence and Rabi oscillations at room temperature. Spin dynamics studies indicate that Ni–O bonds stretching vibration, a local vibrational mode, is crucial for spin–lattice relaxation at high temperatures above 150 K via spin–phonon coupling. This local mode is likely also responsible for the first observation of phonon-induced temperature-dependent Rabi frequency in electron spin qubits. Our simple yet effective bottom-up design strategy provides an approach to developing high-performance spin qubits based on 2D spin arrays. Precisely controlling the spin concentration in 2D c-MOFs and exploring the concentration-dependent spin dynamics remain the focus of our future efforts.

## Supporting Information Available

Experimental methods, characterization data, NMR spectra, TEM images, SEM images, XPS spectra, DFT calculations, conductivity measurements, and EPR measurements (PDF)
