# Supplementary material for: Rational Construction of Layered Two-Dimensional Conjugated Metal–Organic Frameworks with Room-Temperature Quantum Coherence
Source: J Am Chem Soc. 2025 Feb 27;147(10):8778–84. doi: 10.1021/jacs.4c18681 (PMC11912308; doi:10.1021/jacs.4c18681)
Supplement: Supplementary file 1 — ja4c18681_si_001.pdf [file ja4c18681_si_001.pdf]

Supporting Information

Rational Construction of Layered Two-Dimensional Conjugated Metal-Organic Frameworks with Room-Temperature Quantum Coherence

Yang Lu,[1,2,3,11+] Yubin Fu,[2+] Ziqi Hu,[4,5*] Shiyi Feng,[3] Morteza Torabi,[3] Lei Gao,[6] Shuai Fu,[3,6] Zhiyong Wang,[2,3] Chuanhui Huang,[3] Xing Huang,[2] Mingchao Wang,[2,3,12] Noel Israel,[7] Evgenia Dmitrieva,[7] Hai I. Wang,[6,8] Mischa Bonn,[6] Paolo Samorì,[1] Renhao Dong,[9,10*] Eugenio Coronado,[4*] Xinliang Feng[2,3*]

[1]Université de Strasbourg, CNRS, ISIS, UMR 7006, 8 Alleé Gaspard Monge, 67000 Strasbourg, France.

[2]Max Planck Institute of Microstructure Physics, 06120 Halle (Saale), Germany.

[3]Center for Advancing Electronics Dresden & Faculty of Chemistry and Food Chemistry, Technische Universität Dresden, 01067 Dresden, Germany.

[4]Instituto de Ciencia Molecular (ICMol), Universitat de València, 46980 Paterna, Spain.

[5]Department of Materials Science and Engineering, CAS Key Laboratory of Materials for Energy Conversion, Anhui Laboratory of Advanced Photon Science and Technology, University of Science and Technology of China, 230026 Hefei, China.

[6]Max Planck Institute for Polymer Research, 55128 Mainz, Germany.

[7]Leibniz Institute for Solid State and Materials Research, 01069 Dresden, Germany.

[8]Nanophotonics, Debye Institute for Nanomaterials Science, Utrecht University, Princetonplein 1, 3584 CC Utrecht, The Netherlands.

[9]Department of Chemistry, The University of Hong Kong, Hong Kong 999077, China.

[10]Materials Innovation Institute for Life Sciences and Energy (MILES), HKU-SIRI, Shenzhen 518048, China.

[11]MOE Key Laboratory of Low-grade Energy Utilization Technologies and Systems, School of Energy & Power Engineering, Chongqing University, 400044 Chongqing, China.

[12] School of Advanced Materials, Peking University, Shenzhen Graduate School, 518055 Shenzhen, China.

*huziqi@ustc.edu.cn; rhdong@hku.hk; eugenio.coronado@uv.es; xinliang.feng@tu-dresden.de

+Y.L. and Y.F. contributed equally to this work

Table of Contents:

Section S1 Materials and methods

Section S2 Synthetic procedures and NMR spectra for all compounds

Section S3 Supplementary Figures and tables

Section S1 Materials and Methods

Materials

All solvents, reagents, and chemicals were purchased from commercial suppliers, such as Sigma-Aldrich, TCI, and ABCR GmbH. and used without further purification unless specially addressed.

Methods

Nuclear magnetic resonance (NMR) spectroscopy was recorded on Bruker AV-II 300 spectrometer operating at 300.1 MHz and Bruker AV-III 600 spectrometer operating at 600 MHz for ^{1}H NMR and 151 MHz for ^{13}C NMR. Chemical shifts are given in ppm relative to TMS.

Fourier transform infrared (FT-IR) spectroscopy was performed on a Bruker Optics ALPHA-E spectrometer with a universal Zn-Se ATR (attenuated total reflection) accessory in the 400−4000 cm^{-1}.

High-resolution MALDI-TOF mass spectrometry was recorded on a Bruker Autoflex Speed MALDI TOF MS (Bruker Daltonics, Bremen, Germany) with trans-2-[3-(4-tert-Butylphenyl)-2-methyl-2-propenylidene] malononitrile (DCTB) as the matrix. The instrument is equipped with an Agilent Series 1200 HPLC binary pump and Autosampler, using Mass Hunter software.

Ultraviolet-Visible (UV/vis) absorption was measured on an Agilent Cary 5000 UV-VIS-NIR spectrophotometer at room temperature.

Powder X-ray diffraction (PXRD) patterns were obtained on an X-ray diffractometer (Dectris Mythen 1K Strip Detector, Stoe Stadi-P)) using Cu-Kα radiation (λ = 0.154 nm) at 40 kV and 30 mA at room temperature. The as-obtained powder samples were measured in transmission geometry.

Scanning electron microscopy (SEM) was recorded on Zeiss Gemini S4500; **Transmission electron microscope (TEM)** was recorded on JEOL JEM F200.

Thermal gravimetric analysis (TGA) was characterized using Thermal gravity analyses (TGA) carried out on a TA Instrument Q600 analyzer under N_2 atmosphere with a heating rate of 5 ^{o}C/min in a ceramic crucible. Before the measurement, the powder sample was treated in a supercritical CO_2 dryer for 4 hours and then activated at 90 °C overnight. And the *in-situ* activation (100 °C for 1 h) was conducted during the TGA measurement.

Gas adsorption

Nitrogen sorption isotherms were measured on a Micrometrics ASAP 2020-M adsorption analyzer at liquid nitrogen temperature, and the surface area was calculated based on the adsorption curve according to the Brunauer-Emmett-Teller (BET) theory.

Nitrogen sorption measurements were conducted at 77 K on a Quantachrome volumetric analyzer. Pore size distributions were calculated using the quenched solid density functional theory (QSDFT) equilibrium model. Before the measurement, the powder sample was activated at 90 °C overnight.

X-ray photoelectron spectroscopy (XPS) was conducted on a Kratos AXIS Supra/Ultra Photoelectron Spectrometer under an ultrahigh vacuum of about 3×10^{-9} Torr with an unfiltered He I gas discharge lamp source (21.22 eV) and a monochromatic Al Kα source (1486.7 eV, θ = 90°, operated at 14 kV and 15 mA), respectively. The instrumental energy resolution for UPS and XPS were 0.1 eV and 0.5 eV, respectively. For sample preparation, all films were deposited on heavily doped n-type Si wafers in a N_2 glove box and transferred through a transport system without air exposure into the spectrometer analysis chamber.

Electrical conductivity

The pressed pellets were prepared by adding 8 mg samples onto a polymer film in a split sleeve and pressing under pressure at room temperature. The pellets were heated at 150 °C in a vacuum for 2 hours for complete desolvation. Then the pressed pellets were taken out and the thickness was measured. Then, four probes of silver wires were placed onto the top of the pressed pellets using conductive silver plastic. The probe was transferred onto the probe station. Then, the device was kept in air for 1 h to keep the complete drying of the paste. The electrical conductivities of the samples in this work were determined in the parallel four-probe geometries using a commercial Lakeshore Hall System (9700A). The activation energy was obtained through the Arrhenius equation: $\sigma(\mathrm{T}) = \sigma_0 \exp(-E_a/k_B T)$, σ_0 is the pre-factor and k_B is the Boltzmann's constant.

Time-resolved terahertz spectroscopy (TRTS)

TRTS was employed to characterize the charge transport properties of the samples. In the TRTS measurements, an ultrashort ~50 femtosecond pump pulse optically injects charge carriers into the sample via above-bandgap excitation (3.1 eV photon energy). A time-delayed single-cycle THz pulse (~1 ps duration) interacts with the photogenerated charge carriers, probing their transport properties in a contact-free manner. The pump-induced relative attenuation of the THz electric field is linearly proportional to the photoconductivity. Driven by a commercial Ti:sapphire laser amplifier system, 1.55 eV ultrashort laser pulses with a repetition rate of 1 kHz and duration of ~50 fs were generated. The 3.1 eV pulsed laser used for photoexcitation was generated by frequency doubling the 1.55 eV pulsed laser through a BiB_3O_6 crystal. THz generation and detection were achieved by optical rectification and free-space electro-optic sampling, respectively. The measurements were performed in a N_2 environment at room temperature. The frequency-resolved photoconductivity was

analyzed by the Drude-Smith (DS) model. In the DS model, charge carriers experience preferential back scatterings, following:

$$\Delta\sigma(\omega) = \frac{\omega_p^2 \varepsilon_0 \tau}{1 - i\omega\tau}\left(1 + \frac{c}{1 - i\omega\tau}\right)$$

Where ω_p is the plasma frequency, ε_0 is the vacuum permittivity, τ is the DS charge scattering time, and c (ranging from –1 to 0) is the backscattering parameter describing the degree of suppression of conductivity in the DC limit.

EPR measurements

The powder samples were heated at 150 °C in a vacuum for 2 hours to ensure complete desolvation. The samples were then placed in a quartz EPR tube, purged with He, and sealed under vacuum to exclude the influence of H_2O, O_2, and other impurities. A continuous helium gas flow cryostat was used for measurements at low temperatures. CW spectra were measured on a Bruker Elexsys E580 spectrometer operating in the X-band (ω = 9.47 GHz). Microwave power and modulation magnetic field were carefully adjusted in order not to saturate the ESR signal. The free radical TEMPO was used as a standard spin-counting reference. The theoretical minimum distance between two spin centers is approximately 3.4 Å in both Ni_3HHTH_2 and Ni_3HATI_2. However, the average distance between spin centers, calculated based on spin concentration and chemical structure, is approximately 49 Å in Ni_3HHTH_2 and 106 Å in Ni_3HATI_2.
Pulsed EPR data were collected on the same system by a 9.70 GHz cavity. The low-temperature environment was achieved by Oxford Instruments CF935 and ITC503 temperature controller. The signal of the pulsed-EPR experiments was collected by integrating the Hahn echo (π/2-τ-π-τ-echo). The T_1 values were measured by the inversion recovery method (π-T-π/2-τ-π-τ-echo) with 4-step phase cycling. The T_2 values were obtained by increasing the τ value of the Hahn echo sequence with 2-step phase cycling. The π/2 and π pulse lengths in EDFS, T_1 and T_2 measurements were 16 and 32 ns, respectively, with 10 dB attenuation of the microwave power. The nutation experiments were carried out with standard sequence (t_p-T-π/2-τ-π-τ-echo), where T > $5T_2$.
The inversion recovery and Hahn echo decay curves were fitted by the standard monoexponential functions as follows to extract T_1 and T_2.

$$I = I_0 + k_1 exp[-(\tau/T_1)] \text{ and } I = I_0 + k_2 exp[-(2\tau/T_2)]$$

where I is echo intensity, k_1 and k_2 represent the pre-factor, and I_0 denotes the baseline drift.

Pawley refinement

The unit cells of the models were refined in the 2θ range 2.5-40° with the experimentally obtained PXRD pattern of Ni_3HHTH_2 and Ni_3HATI_2 2D c-MOFs in the Reflex module of the BIOVA Materials Studio 2020 (20.1.0.5. Copyright © 2019 Dassault Systèmes), with fixed atom coordinates. The obtained structural models were checked for bond length and bond angle consistency in the structure. The Pseudo-Voigt profile function was used to fit the whole profile during the refinement processes.

Modeling and First-principles calculations

In order to screen among various tacking order of the 2D c-MOFs a highly efficient parallel CP2K/Quickstep package.[1,2] Density functional theory was applied within the generalized gradient approximation (GGA), using PBEsol functional.[3] Basis set DZVP-MOLOPT-SR-GTH,[4] which is optimized for calculating molecular properties in gas and condensed phase, was applied for all atoms in the studied systems. For reducing computational cost, Gaussian and Plane-Wave (GPW) methods were used.[5,6] This method uses an atom-centered Gaussian-type basis to describe the wave functions and an auxiliary plane wave basis to describe the electron density. Only the valence electrons were explicitly treated. Their interaction with the remaining ions is described using the pseudopotentials of Goedecker−Teter−Hutter (GTH).[7,8] To take into account the dispersion interactions correctly the DFT-D3(BJ) method was used.[9] The electronic properties (band structures, DOS) were performed in the Vienna Ab initio Simulation Package (VASP) with the pre-optimized structure.[7,8] The electronic exchange-correlation function was treated by the Perdew, Burke and Ernzerhof (PBE) approach with D3BJ dispersion correction.[9] The energy cutoff of the plane waves was set to 600 eV. The energy convergence criterion in the self-consistent calculations was set to 10^{-6} eV. The PBE XC-functional was combined with a simplified rotationally invariant LSDA+U approach introduced by Dudarev et al.[10] so that only the difference U−J was applied in the calculations. The U-J value was set to 3 eV as it has been shown to be a reasonable value for the electronic properties of Ni-containing MOFs.[11] Monkhorst-Pack Gamma-centered grid with 2×2×1 dimension was used for K-point sampling of the Brillouin zone for the monolayer during the SCF calculations. In the computational protocol for the three-dimensional (3D) stacking of the studied MOFs, the K-point grid dimension was changed to 1×1×4 for the SCF calculations. Vaspkit was used to generate the k-path for the band structure calculations (https://vaspkit.com). The MOF monolayer was modeled by adding a large vacuum space, 15 Å, in the direction normal to the monolayer. All the models were subject of full geometry optimization (cell parameters and ionic positions). To identify the vibration modes of the MOF model compounds, DFT calculation was performed using the Gaussian 16 program.[12] The B3LYP functional with Grimme’s D3 correction (Becke–Johnson damping)[13] was used for geometry optimization and frequent calculation in the ground state. The 6-31G(d) basis set was used. All geometry optimization was done in the gas phase.

Section S2 Synthetic procedures

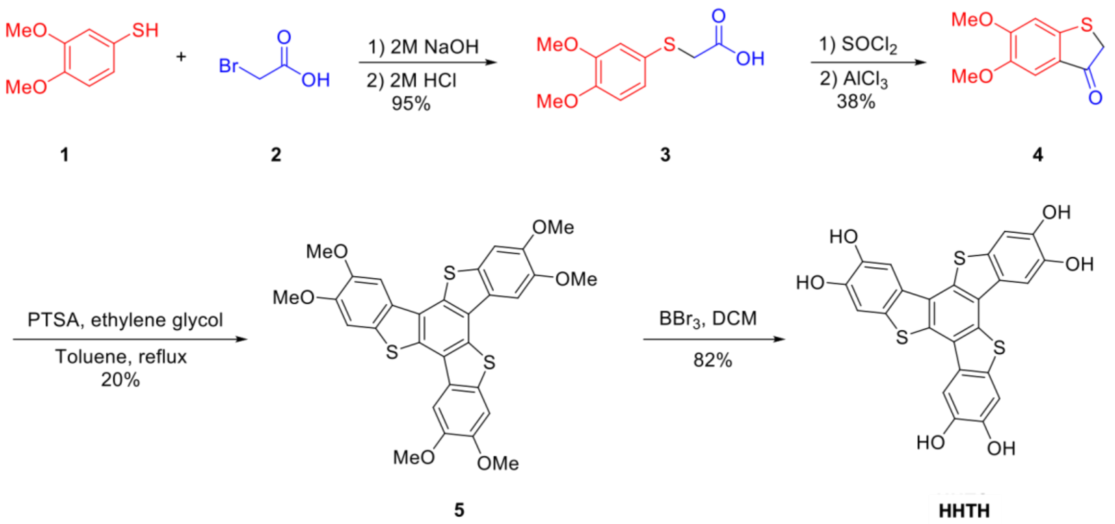

General Procedure:

The synthesis approaches of **compound 3**, **compound 4**, and the **HATI** ligand have been reported in previous research.[14]

Compound 5

Initially, **4** (0.27 g, 1.28 mmol, 1 eq), PTSA (0.072 g, 0.38 mmol, 0.3 eq), and ethylene glycol (0.014 g, 0.22 mmol, 0.17 eq) were added to a two-neck flask and degassed for three times. Anhydrous toluene (20 mL) was then added, and the mixture was refluxed for 24 h. After cooling to room temperature, the solvent was removed under reduced pressure. Ethanol (10 mL) and DCM (15 mL) were subsequently added, and the mixture was refluxed for 30 min to recrystallize the crude products. The solution was cooled to room temperature to allow the precipitate to form. The solid was filtered and washed with ethanol to give the pure **5** with light pink solids and a 20% yield. ^{1}H NMR (300 MHz, $CDCl_3$) δ 7.66 (s, 1H), 7.22 (s, 1H), 4.12 (s, 3H), 4.02 (s, 3H).

HHTH

Initially, **5** (0.07 g, 0.12 mmol, 1 eq) was added to a two-neck flask and degassed three times. Anhydrous DCM (15 mL) was then added, and the flask was cooled in an ice bath. BBr_3 (0.72 mmol, 6 eq) was added, and the reaction was stirred at room temperature for 12 h. The reaction was quenched by adding MeOH (10 mL) at 0 °C and stirring for 15 min to precipitate the product. The solid was centrifuged and then washed sequentially with H_2O (10 mL) and MeOH (10 mL) to give the product as a light gray solid with 82% yield. ^{1}H NMR (300 MHz, DMSO) δ 9.63 (d, J = 3.3 Hz, 2H), 7.92 (s, 1H), 7.53 (s, 1H). ^{13}C NMR (76 MHz, DMSO) δ 146.79, 145.72, 129.51, 129.05, 127.13, 126.66, 109.48, 109.16.

Ni_3HATI_2 synthesis:

$Ni(OAc)_2{\cdot}4H_2O$ (1.5 eq) and NH_4OAc (150 eq) in the solvent mixed with 2 mL DMSO and 2 mL H_2O were preheated at 65 °C, to which was added a solution of 5 mg (1 eq.)

of HATI·6HCl in 1 mL of DMSO. This mixture was heated in a 20 mL open glass vial with stirring for 2 hours at 65 °C. The resulting black powder was filtered, washed with a copious amount of water, DMF, and acetone, and dried under vacuum at room temperature (89 % yield). Elemental analysis for Ni_3HATI_2 ($Ni_3(C_{24}H_{15}N_9)_2$): Calculated: C: 55.70%; H: 2.92%; N: 24.36%. Found: C: 55.60%; H: 2.97%; N: 24.30%.

Ni_3HHTH_2 synthesis:

$Ni(acac)_2$ (1.5 eq), HHTH monomer (5 mg, 1 eq), and NH_4OAc (150 eq) in 0.5 mL DMF and 1.5 mL H_2O. This mixture was heated in a 20 mL glass vial without stirring for 24 hours at 85 °C. The resulting powder was filtered, washed with a copious amount of water, DMF, and acetone, and dried under a vacuum to give a deep green product with 96 % yield. Elemental analysis for Ni_3HHTH_2 ($Ni_3(C_{24}H_6O_6S_3)_2$): Calculated: C: 50.17%; H: 1.05%; S: 16.74%. Found: C: 50.11%; H:1.08%; S: 16.69%.

NMR spectra for all compounds

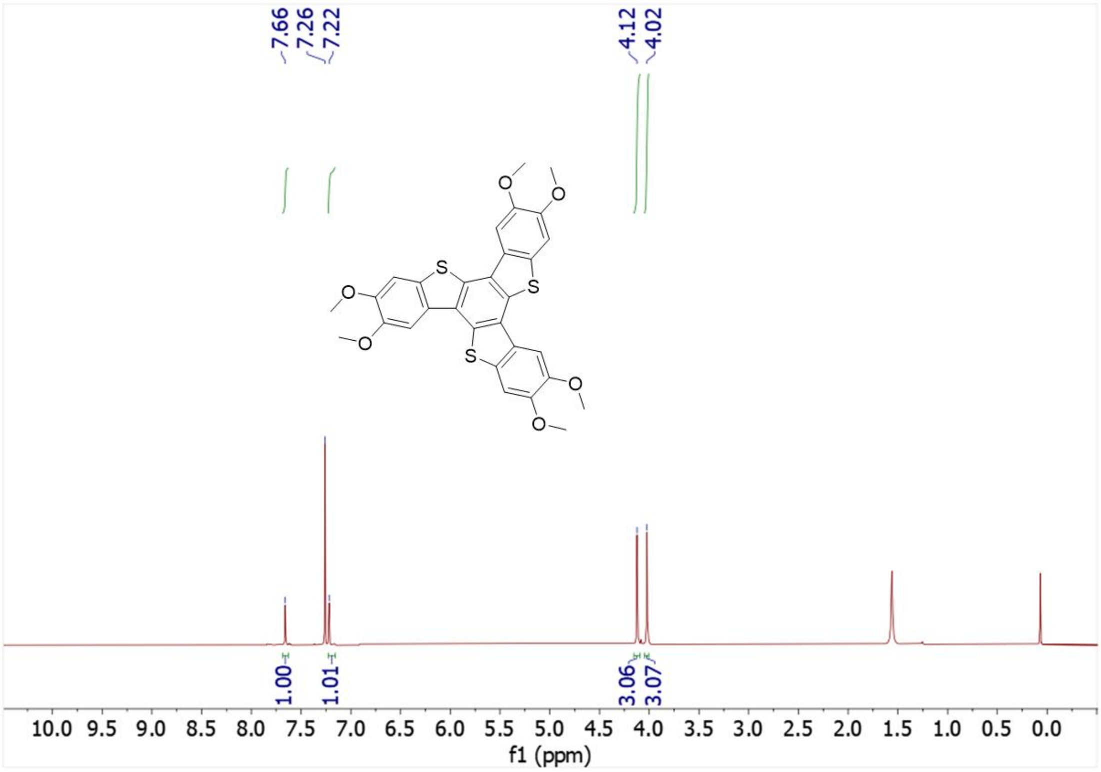

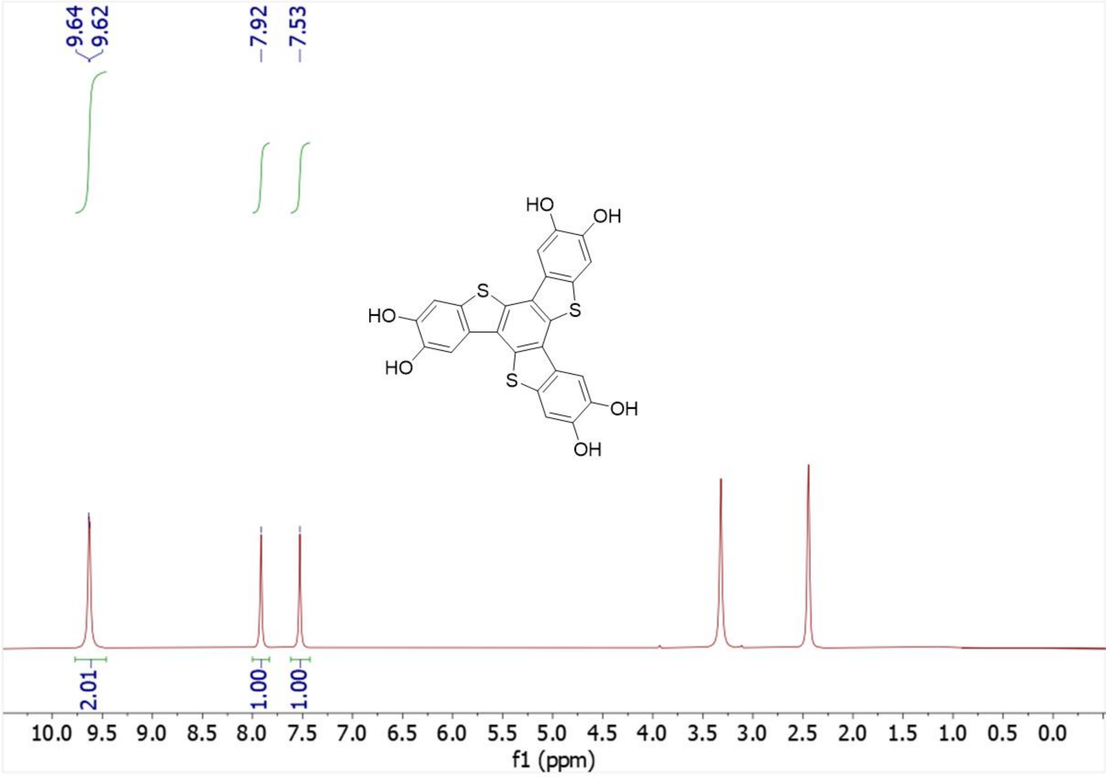
9.64
9.62
7.92
7.53
HO
OH
S
S
HO
HO
S
OH
OH
2.01
1.00
1.00
10.0 9.5 9.0 8.5 8.0 7.5 7.0 6.5 6.0 5.5 5.0 4.5 4.0 3.5 3.0 2.5 2.0 1.5 1.0 0.5 0.0
f1 (ppm)

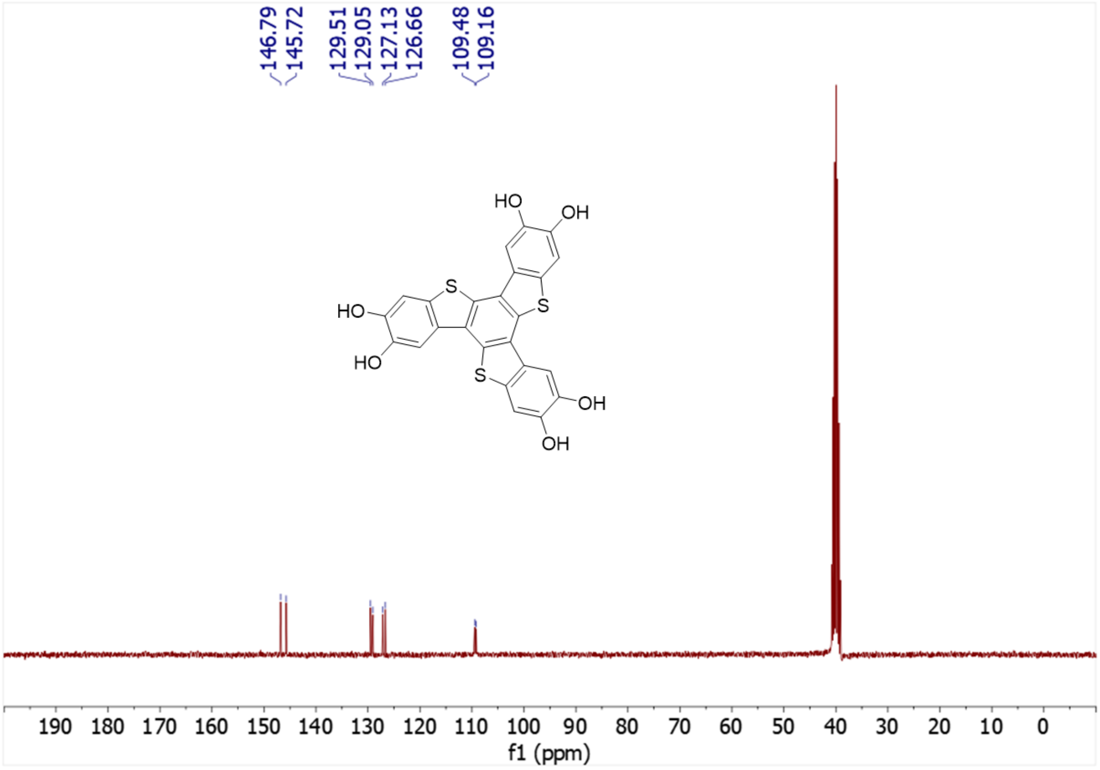
146.79
145.72
129.51
129.05
127.13
126.66
109.48
109.16
HO
OH
S
S
HO
HO
S
OH
OH
190 180 170 160 150 140 130 120 110 100 90 80 70 60 50 40 30 20 10 0
f1 (ppm)

Section S3 Supplementary Figures

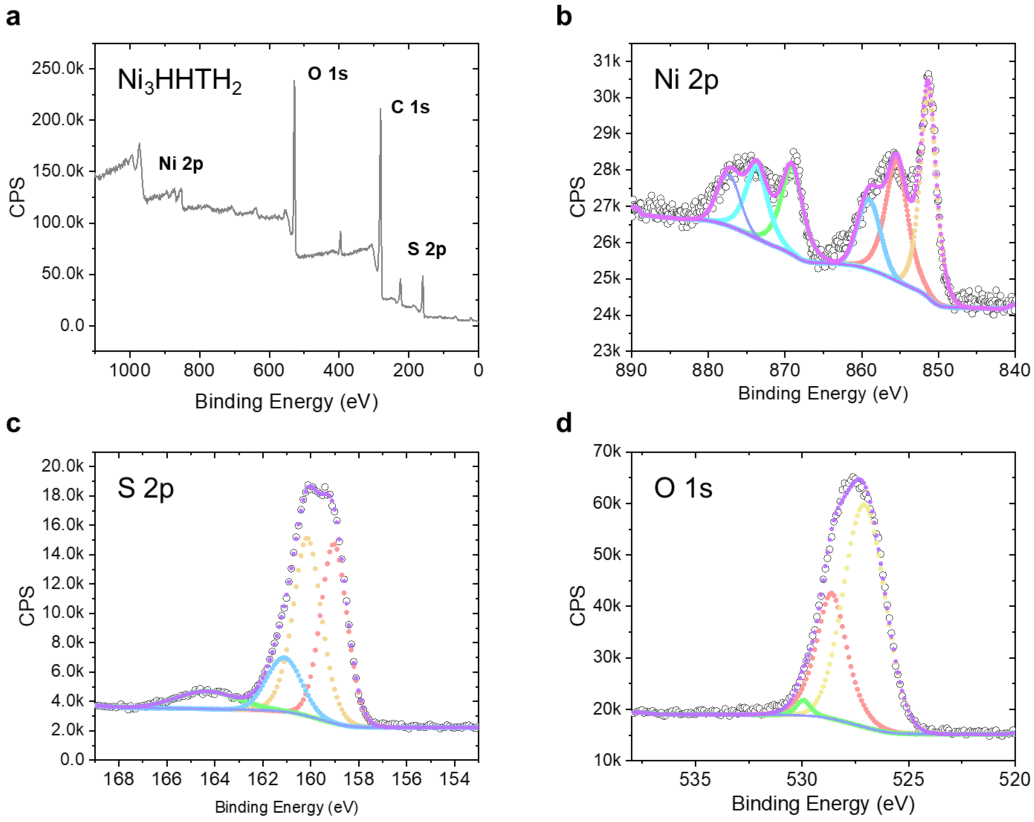

Figure S1. XPS High-resolution X-ray photoelectron spectroscopy spectrum for Ni_3HHTH_2 sample. (a) X-ray photoelectron spectroscopy survey spectrum, high-resolution spectrum for (b) Ni (2p), (c) S (2p), and (d) O (1s) of Ni_3HHTH_2.

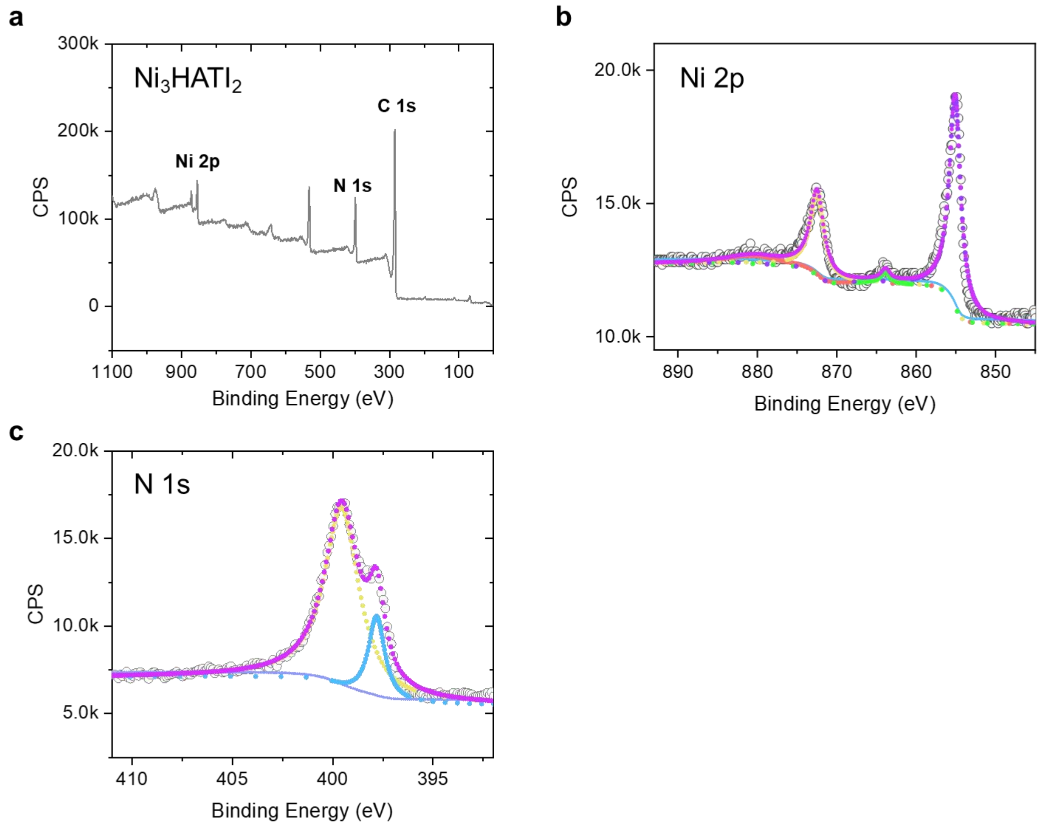

Figure S2. XPS High-resolution X-ray photoelectron spectroscopy spectrum for Ni_3HATI_2 sample. (a) X-ray photoelectron spectroscopy survey spectrum, high-resolution spectrum for (b) Ni (2p) and (c) N (1s) of Ni_3HATI_2.

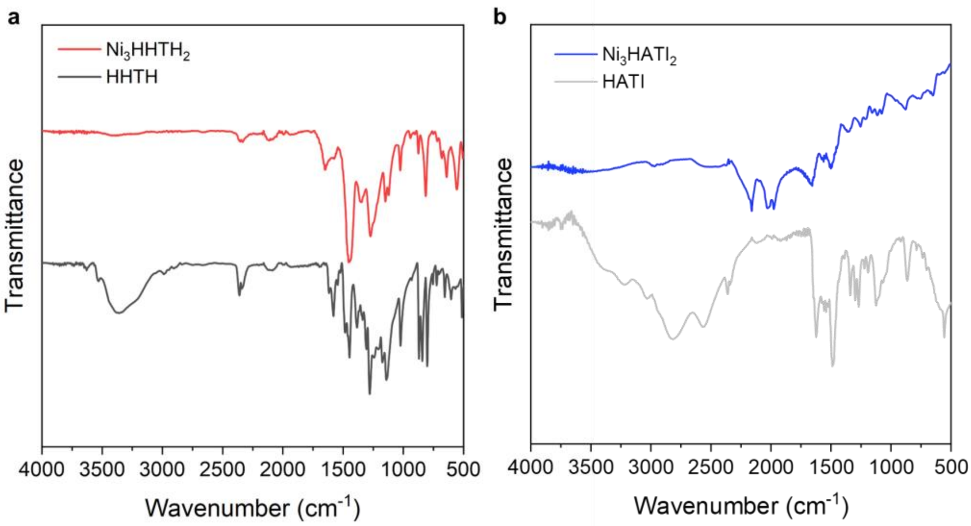

Figure S3. FT-IR Fourier-transform infrared spectra of (a) HHTH and Ni_3HHTH_2, (b) HATI and Ni_3HATI_2. The FT-IR spectra reveal the disappearance of the O-H and N-H stretching vibration bands from the conjugated ligands HHTH and HATI, which further demonstrates the efficient coordination polymerization.

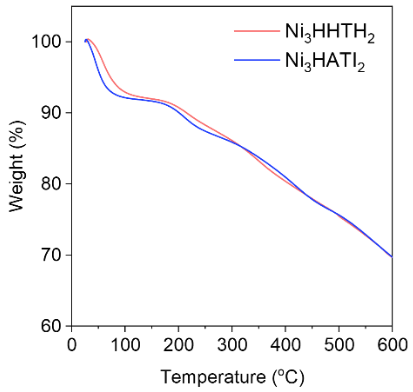

Figure S4. Thermogravimetric Analysis (TGA). TGA revealed that Ni_3HHTH_2 and Ni_3HATI_2 decompose above 200 °C. The weight loss before 100 °C of the samples could not be totally excluded. The reason for this phenomenon might be originated from the strong hydrophilicity of the NiO_4 and $Ni[NH]_4$ nodes which leads to the rapid water uptake of the samples during the weighing and transfer process.

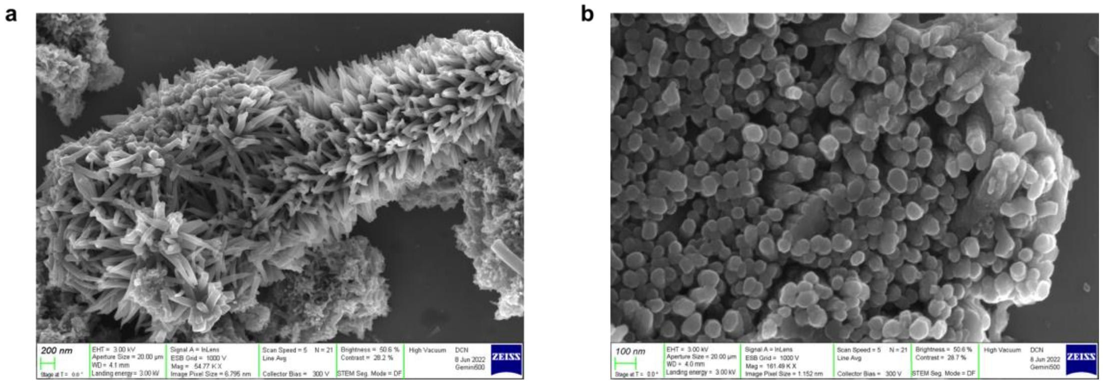

Figure S5. SEM images of (a) Ni_3HHTH_2 and (b) Ni_3HATI_2. Both samples show the rod-like crystals.

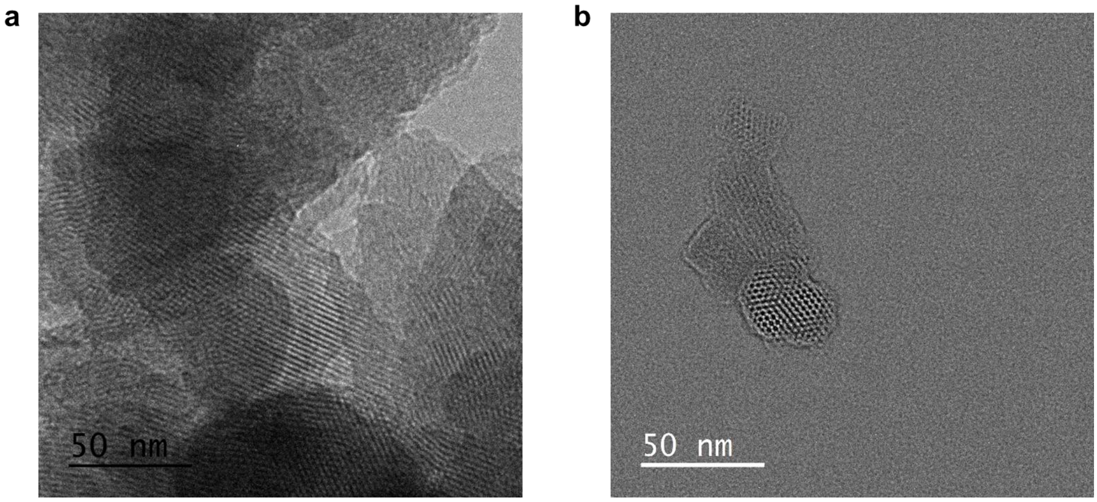

Figure S6. HRTEM images of Ni_3HATI_2: (a) imaged normal to the *c* direction and (b) imaged along the *c* direction.

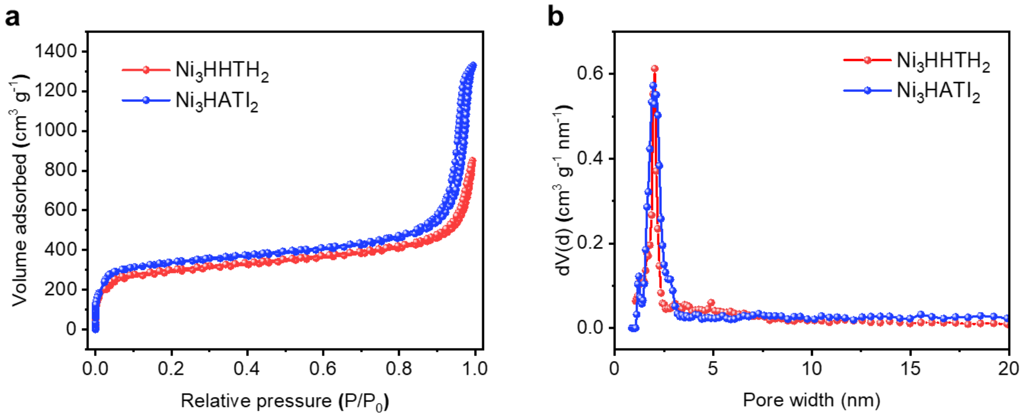

Figure S7. Porosity properties of the samples. (a) N_2 adsorption/desorption isotherms at 77.3 K of Ni_3HHTH_2 and Ni_3HATI_2. (b) the corresponding pore size distribution of Ni_3HHTH_2 and Ni_3HATI_2 (calculated using the NLDFT method). Both materials showed the similar BET surface area and the pore distribution, suggesting the both 2D c-MOFs are isostructures.

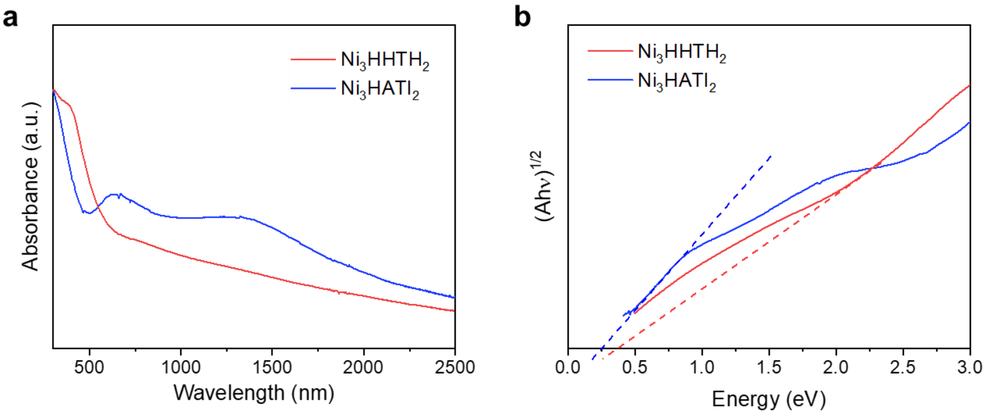

Figure S8. Tauc plots of for Ni_3HHTH_2 and Ni_3HATI_2. Dashed lines indicate the best linear fits to the absorption edges. Tauc plot is mainly based on the formula proposed by Tauc, Davis, and Mott et al.: $(\alpha h\nu)^{1/n} = B(h\nu - Eg)$. Based on the above formula, (αhν)1/n is linear with hν, which can be used to estimate E_g. The optical gaps estimated from the Tauc plots of Ni_3HHTH_2 and Ni_3HATI_2 are 0.40 and 0.25 eV, respectively.

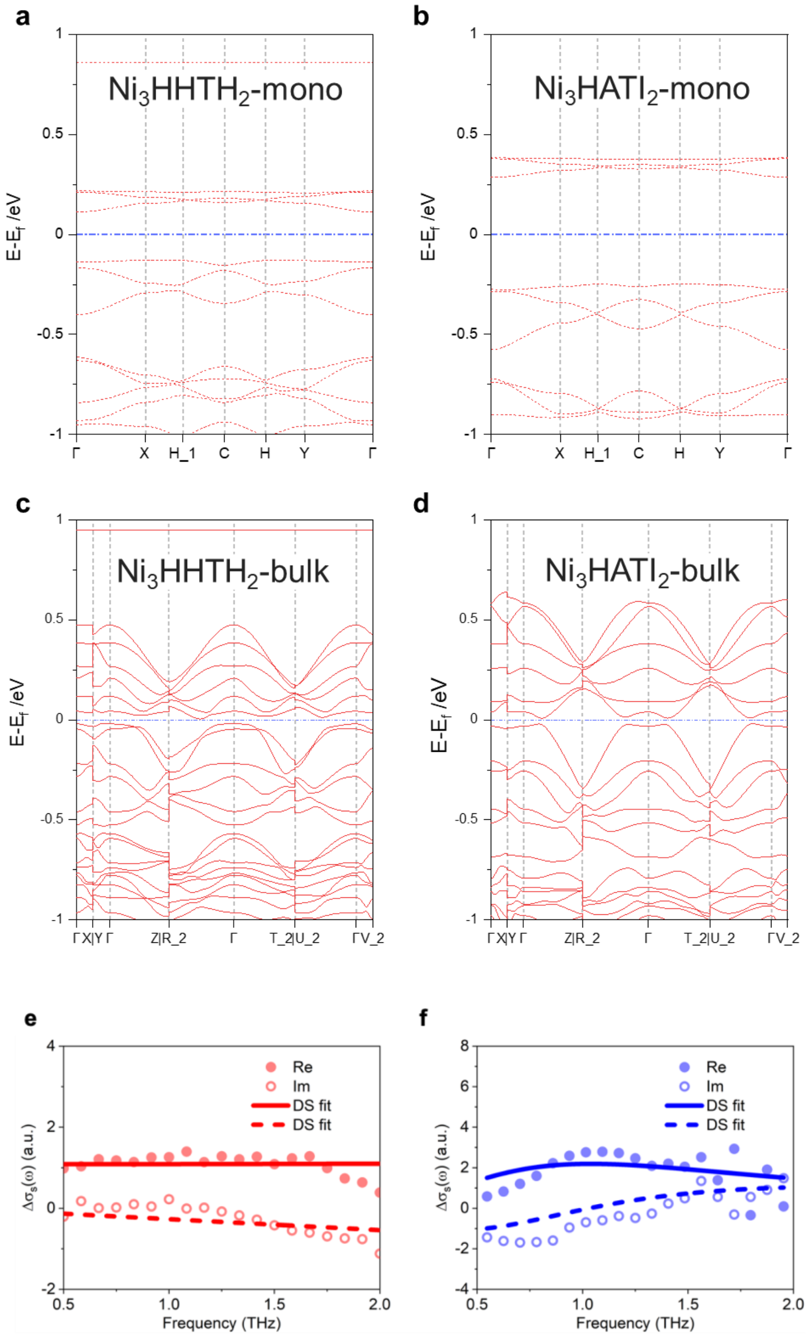

Figure S9. Modeling of the electronic structures of Ni_3HHTH_2 and Ni_3HATI_2. The calculated band structures of monolayer **a** Ni_3HHTH_2, **b** Ni_3HATI_2. The calculated band structures of bulk **c** Ni_3HHTH_2, **d** Ni_3HATI_2. In all plots, the Fermi energy is set to 0 eV. Frequency-resolved complex photoconductivity of **e** Ni_3HHTH_2 and **f** Ni_3HATI_2 measured at 0.5 ps after the maximum photoconductivity. The solid and dashed line correspond to the DS fit to the real and imaginary parts of the complex photoconductivity, respectively.

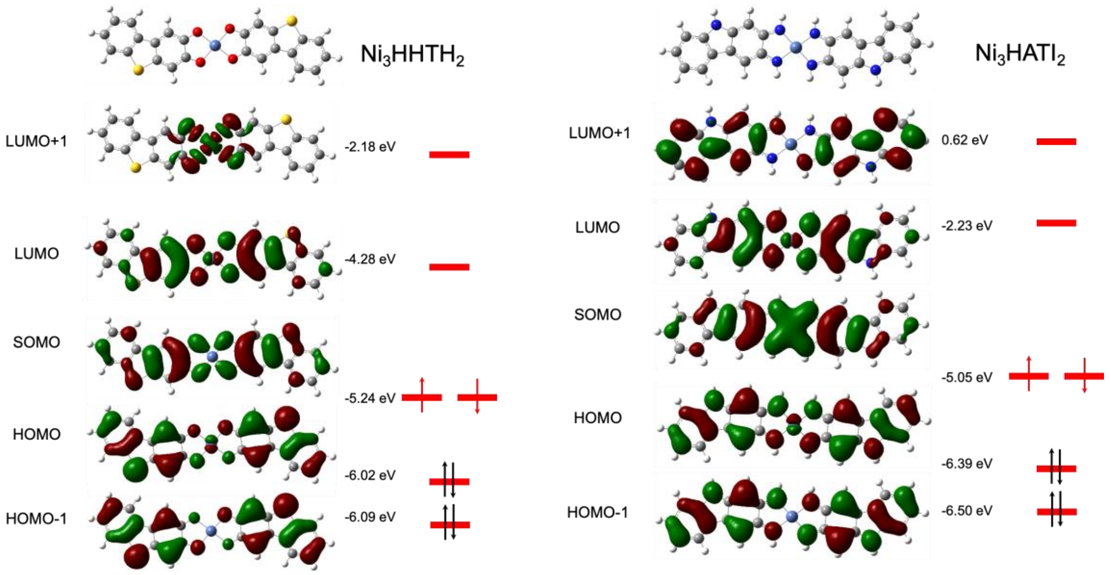

Figure S10. The optimized geometries and frontier orbitals of Ni_3HHTH_2 and Ni_3HATI_2 mono nodes, respectively, in the open-shell singlet state calculated by cam-b3lyp/def2svp.

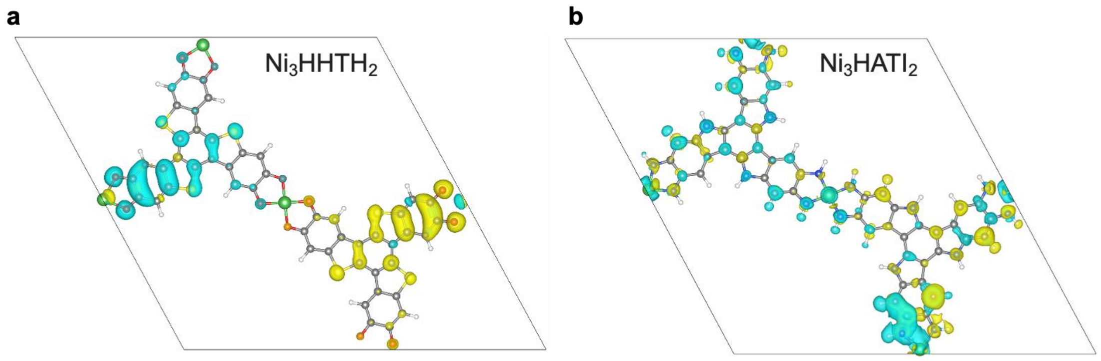

Figure S11. DFT calculation of the spin distribution of monolayer (a) Ni_3HHTH_2 and (b) Ni_3HATI_2.

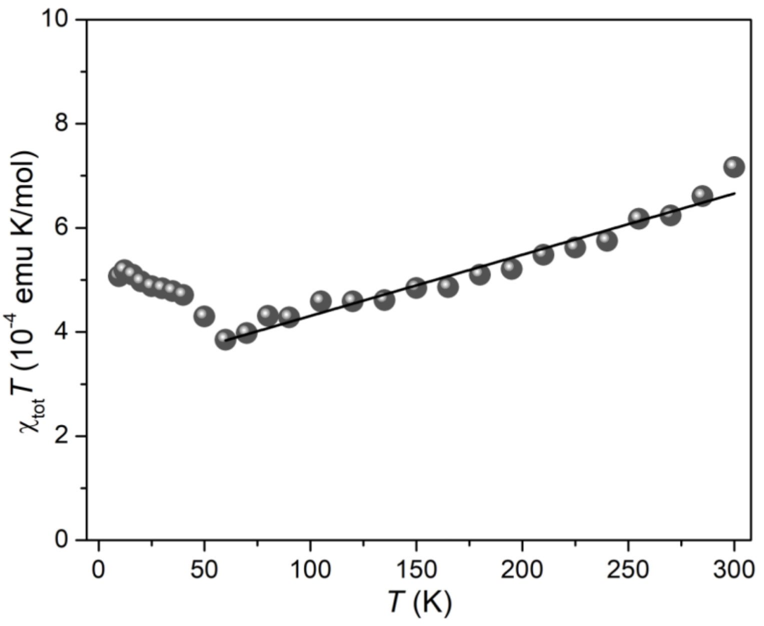

Figure S12. $\chi_{tot}T$ versus temperature (T) plot for the spin susceptibility (χ_{tot}) obtained from the double integration of the variable-temperature CW-EPR spectra of Ni_3HHTH_2. The solid line is the best-fit at the temperature range of 50-300 K considering as a sum of Pauli and Curie susceptibilities: $\chi_{tot}T = \chi_{Pauli}T + C$, where the C is Curie constant, giving rise to $C = 3.1\times10^{-4}$ emu K/mol and $\chi_{Pauli} = 1.2\times10^{-6}$ emu/mol.

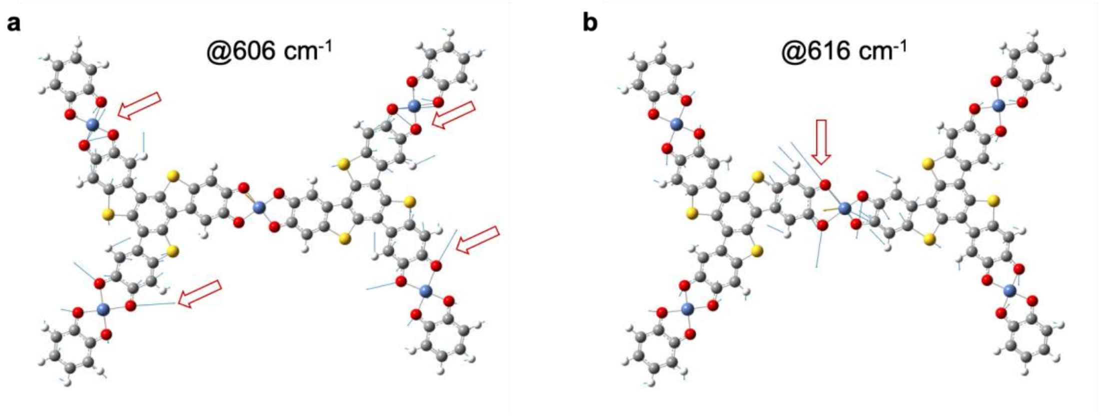

Figure S13. DFT theoretically calculated infrared vibration mode trajectories of Ni_3HHTH_2 fragments at (**a**) 606 cm^{-1} and (**b**) 616 cm^{-1} excitation energies

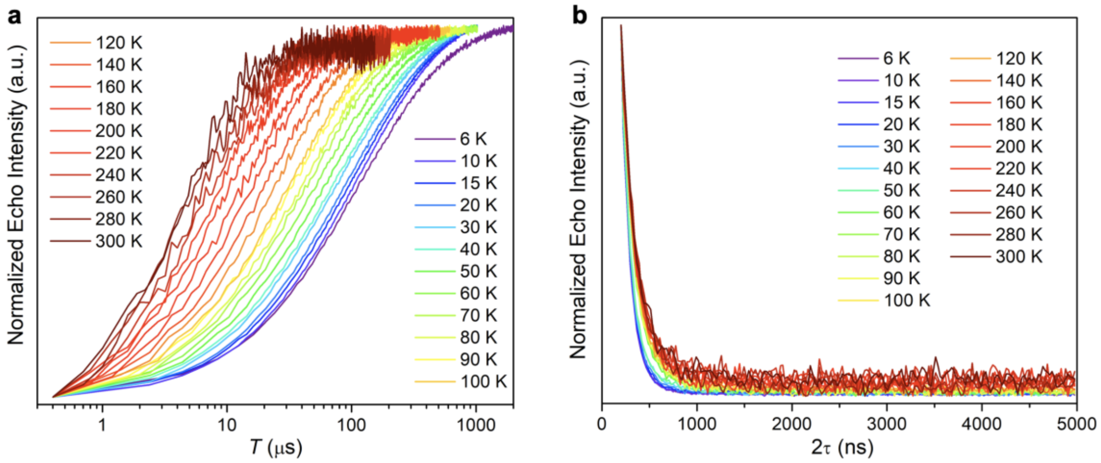

Figure S14. Spin echo inversion recovery (**a**) and decay curves (**b**) for Ni_3HHTH_2 at different temperatures, from which T_1 and T_2 are extracted using exponential fits.

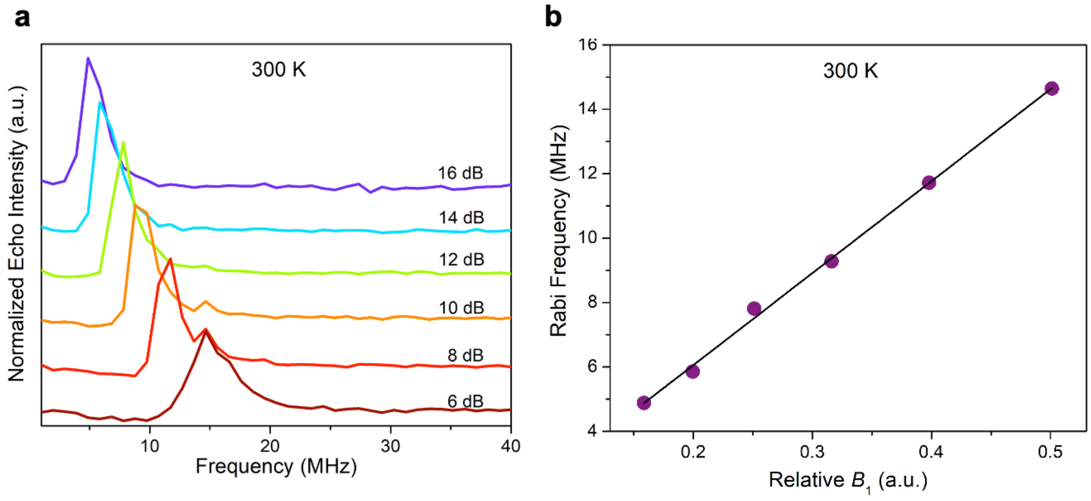

Figure S15. Fast Fourier transform (FFT) analyses of Ni_3HHTH_2 measured at 300 K using different attenuations of the microwave power (**a**), and the relationship between Rabi frequency and the relative magnetic field B_1 of the applied microwave pulse (**b**). The black line is the linear fit to the data (purple circles).

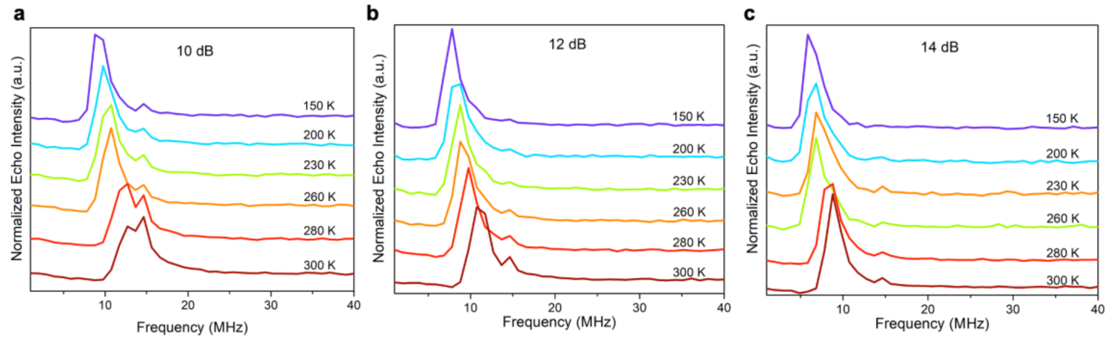

Figure S16. Fast Fourier transform (FFT) analyses of Ni_3HHTH_2 measured at different temperatures (150-300 K) using 10 dB (**a**), 12 dB (**b**), and 14 dB (**c**) attenuations of the microwave power.

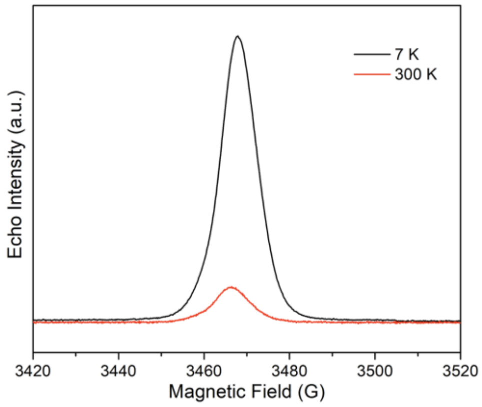

Figure S17. Echo-detected field-sweep (EDFS) spectra of Ni_3HHTH_2 measured at 7 K and 300 K.

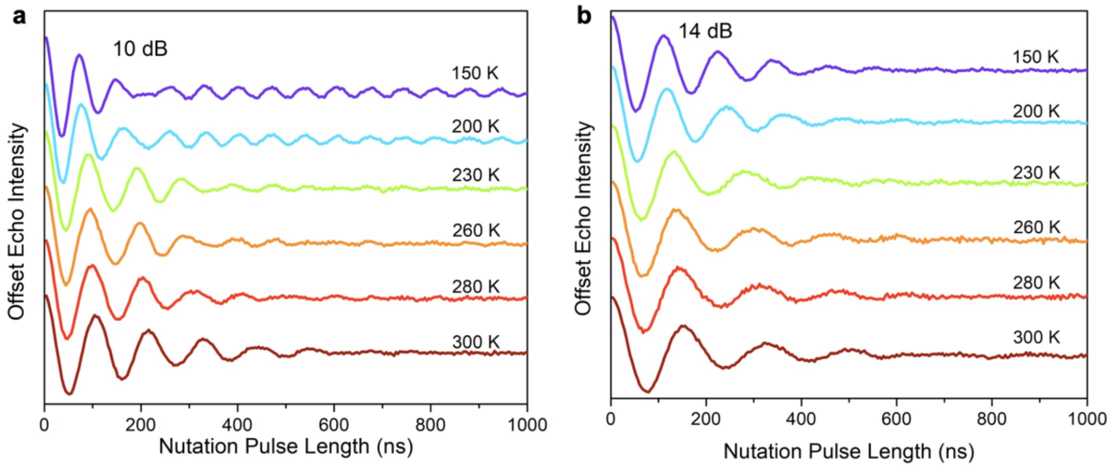

Figure S18. Normalized echo intensity in nutation experiments on Ni_3HHTH_2 using 10 dB (**a**) and 14 dB (**b**) attenuation powers of the applied microwave at 150-300 K.

Table S1 The summary of unit cell parameters of both 2D c-MOFs

	a	*b*	*c*	*α*	*β*	*γ*
Ni_3HHTH_2	27.46 Å	26.95 Å	6.52 Å	90.9°	89.4°	118.4°
Ni_3HATI_2	27.28 Å	26.89 Å	6.50 Å	89.8°	89.5°	118.3°

Table S2 The summary of unit cell elemental analysis of both 2D c-MOFs

Ni_3HHTH_2	C	H	N	S	Ni_3HATI_2	C	H	N	S
Calculation	50.17%	1.05%	-	16.74%	Calculation	55.70%	2.92%	24.36%	-
Batch 1	50.13%	1.08%	< 0.1%	16.69%	Batch 1	55.59%	2.97%	24.30%	-
Batch 2	50.10%	1.09%	< 0.1%	16.67%	Batch 2	55.62%	2.96%	24.31%	-
Batch 3	50.11%	1.08%	< 0.1%	16.70%	Batch 3	55.57%	2.99%	24.28%	-
Ave.	50.11%	1.08%	-	16.69%	Ave.	55.60%	2.97%	24.30%	-

Atomistic coordinates of optimized MOF structures

Ni_3HHTH_2

C	0.304559	15.253423	1.515152
C	9.021794	9.235397	1.744588
C	4.773694	13.853884	1.570515
C	-3.856336	12.768063	1.563528
C	18.719963	10.957949	1.742192
C	11.392999	10.040846	1.798420
C	5.714175	14.955002	1.590611
C	1.550761	15.919078	1.523271
C	10.388703	8.999628	1.780321
C	3.381828	14.077224	1.538841
C	16.886123	12.641799	1.787906
C	12.979649	4.813444	1.770606
C	12.730006	9.455553	1.816187
C	16.003628	11.575132	1.809533
C	14.005311	10.055777	1.825436
C	18.263385	12.349944	1.748580
C	5.248581	16.280924	1.577369
C	2.908075	15.396548	1.533252
C	-2.469076	12.533777	1.524668
C	0.321249	18.081526	1.544497
C	12.695686	8.027863	1.806538
C	3.872980	16.471387	1.552416
C	15.235911	9.313466	1.812727
C	16.407007	10.183763	1.797355
C	-1.623791	13.636633	1.514620
C	-0.913133	17.355349	1.540039
C	17.781459	9.893932	1.771205
C	13.859859	7.238389	1.803145
C	15.126027	7.903945	1.806006
C	-2.082552	15.010761	1.533651
C	1.522660	17.346169	1.537710
C	-0.955731	15.940518	1.527995
C	-4.357663	14.139512	1.585287
C	13.980222	5.795261	1.785777
C	-3.463671	15.242574	1.567388
C	15.364923	5.392867	1.777859
C	13.351972	3.457305	1.743324
C	0.133785	19.516475	1.566523
C	15.763297	4.062430	1.755434
C	1.090576	20.539478	1.583424
C	-1.267267	19.867906	1.575040
C	14.750403	3.080268	1.735339

C	0.656871	21.874911	1.614166
C	-1.722357	21.181191	1.600925
C	-0.750599	22.209266	1.623216
C	10.964574	11.375827	1.783116
C	8.607618	10.574825	1.722363
C	9.576097	11.650106	1.742725
H	16.550801	13.678043	1.796890
H	8.286709	8.432268	1.728751
H	2.716741	13.215290	1.524468
H	-2.089330	11.513448	1.514221
H	5.951806	17.112978	1.592607
H	18.156701	8.871620	1.764085
H	11.920407	5.058759	1.777459
H	-3.881690	16.248444	1.587017
H	2.161017	20.347010	1.576437
H	16.810636	3.764445	1.751042
H	-2.781852	21.434111	1.606913
H	11.659832	12.214133	1.794893
O	-5.659597	14.237477	1.626676
O	6.978564	14.604336	1.625212
O	19.196792	13.253106	1.717905
O	-4.772965	11.832035	1.591409
O	20.022117	10.825981	1.714311
O	12.517292	2.440827	1.721565
O	14.961303	1.787449	1.706127
O	1.460281	22.918146	1.636657
O	-1.000082	23.495196	1.653093
O	5.344965	12.669303	1.588548
O	7.353402	10.958963	1.676449
O	9.036577	12.847043	1.714378
S	16.446856	6.766293	1.796557
S	14.276915	11.773000	1.845532
S	11.074861	7.411237	1.790235
S	0.111201	13.528568	1.496920
S	3.116653	18.037249	1.552216
S	-2.282181	18.438894	1.552549
Ni	-6.529760	12.533967	1.661943
Ni	13.389396	0.812333	1.679444
Ni	7.188391	12.778138	1.649167
O	-8.269408	13.253106	1.717905
O	-7.444083	10.825981	1.714311
O	14.298372	-0.778231	1.636657
O	11.838009	-0.201181	1.653093
Ni	20.936440	12.533967	1.661943

Ni	0.551306	24.508709	1.679444
C	14.246139	8.268405	5.139008
C	5.528903	14.286431	4.909573
C	9.777004	9.667943	5.083646
C	18.407034	10.753765	5.090633
C	-4.169266	12.563878	4.911969
C	3.157698	13.480981	4.855741
C	8.836523	8.566826	5.063550
C	12.999936	7.602749	5.130890
C	4.161995	14.522199	4.873840
C	11.168870	9.444603	5.115320
C	-2.335425	10.880028	4.866254
C	1.571048	18.708383	4.883555
C	1.820692	14.066274	4.837974
C	-1.452930	11.946696	4.844628
C	0.545386	13.466050	4.828725
C	-3.712687	11.171884	4.905581
C	9.302117	7.240903	5.076792
C	11.642623	8.125279	5.120909
C	17.019774	10.988050	5.129493
C	14.229448	5.440301	5.109664
C	1.855012	15.493965	4.847623
C	10.677718	7.050441	5.101745
C	-0.685213	14.208361	4.841434
C	-1.856310	13.338064	4.856806
C	16.174489	9.885195	5.139541
C	15.463831	6.166478	5.114122
C	-3.230762	13.627896	4.882956
C	0.690838	16.283439	4.851016
C	-0.575330	15.617883	4.848155
C	16.633250	8.511067	5.120510
C	13.028038	6.175658	5.116451
C	15.506429	7.581310	5.126166
C	18.908360	9.382315	5.068874
C	0.570475	17.726567	4.868384
C	18.014368	8.279253	5.086773
C	-0.814226	18.128960	4.876302
C	1.198725	20.064522	4.910837
C	14.416913	4.005352	5.087638
C	-1.212600	19.459398	4.898727
C	13.460122	2.982349	5.070737
C	15.817965	3.653921	5.079121
C	-0.199706	20.441559	4.918822
C	13.893827	1.646917	5.039995

C	16.273054	2.340636	5.053236
C	15.301297	1.312561	5.030945
C	3.586124	12.146000	4.871045
C	5.943080	12.947003	4.931798
C	4.974600	11.871721	4.911436
H	-2.000104	9.843784	4.857271
H	6.263989	15.089560	4.925410
H	11.833956	10.306537	5.129693
H	16.640028	12.008379	5.139940
H	8.598892	6.408849	5.061554
H	-3.606003	14.650208	4.890076
H	2.630291	18.463069	4.876701
H	18.432387	7.273383	5.067144
H	12.389680	3.174817	5.077724
H	-2.259939	19.757383	4.903118
H	17.332550	2.087716	5.047248
H	2.890866	11.307694	4.859268
O	20.210294	9.284350	5.027485
O	7.572133	8.917492	5.028949
O	-4.646095	10.268721	4.936256
O	19.323662	11.689792	5.062752
O	-5.471420	12.695847	4.939849
O	2.033406	21.081000	4.932596
O	-0.410605	21.734378	4.948034
O	13.090416	0.603681	5.017503
O	15.550779	0.026632	5.001068
O	9.205733	10.852524	5.065613
O	7.197295	12.562865	4.977712
O	5.514120	10.674785	4.939783
S	-1.896158	16.755535	4.857604
S	0.273783	11.748827	4.808629
S	3.475837	16.110591	4.863925
S	14.439497	9.993260	5.157241
S	11.434045	5.484579	5.101945
S	16.832878	5.082934	5.101612
Ni	21.080457	10.987860	4.992218
Ni	1.161301	22.709495	4.974717
Ni	7.362307	10.743689	5.004994
O	22.820105	10.268721	4.936256
O	21.994780	12.695847	4.939849
O	0.252326	24.300058	5.017503
O	2.712689	23.723009	5.001068
Ni	-6.385743	10.987860	4.992218
Ni	13.999392	-0.986882	4.974717

Ni_3HATI_2

C	-0.213862	15.852399	5.073370
C	12.660690	2.997235	4.755269
C	1.197881	20.449710	4.998397
C	-4.275411	13.624649	4.955871
C	18.929540	10.334751	4.790772
C	14.668133	4.468496	4.731080
C	2.653200	20.523699	5.009516
C	0.952040	16.637134	5.084360
C	13.240743	4.244109	4.719180
C	0.524225	19.211754	5.024927
C	19.381813	7.876115	4.814832
C	10.791997	8.372415	4.663974
C	14.842896	5.900376	4.699282
C	18.011815	7.700945	4.770029
C	15.921893	6.791467	4.712092
C	19.856623	9.205846	4.829722
C	3.416464	19.338660	5.048076
C	1.273706	18.045160	5.060235
C	-3.740231	14.936681	4.978174
C	2.297605	14.547063	5.113751
C	13.538760	6.483434	4.667615
C	2.719536	18.141894	5.072330
C	15.762376	8.211639	4.695771
C	17.074469	8.799799	4.734331
C	-2.362416	15.063748	5.020050
C	1.095468	13.797908	5.109134
C	17.541163	10.108835	4.738687
C	13.326451	7.858108	4.648368
C	14.459383	8.718410	4.661113
C	-1.482869	13.921032	5.050352
C	2.195079	15.935583	5.107313
C	-0.149694	14.422265	5.087936
C	-3.386112	12.466287	4.984351
C	12.150730	8.669983	4.648043
C	-1.994959	12.629665	5.034941
C	12.608078	10.040165	4.657406
C	9.876418	9.434723	4.702469
C	3.387268	13.620582	5.089106
C	11.737614	11.108498	4.680490
C	4.761550	13.781511	5.047556
C	2.812422	12.299421	5.079353
C	10.357015	10.809882	4.712157

C	5.563275	12.637062	4.986172
C	3.578130	11.144212	5.033640
C	4.982337	11.305515	4.980450
C	15.529349	3.367354	4.776272
C	13.527790	1.895424	4.805273
C	14.971350	2.072421	4.814571
H	17.678410	5.591985	4.806248
H	4.103901	21.922415	4.976874
H	20.078225	7.035345	4.842597
H	11.580216	2.856962	4.747661
H	-0.565476	19.201714	5.014133
H	21.838802	8.883223	4.906582
H	-4.404334	15.800244	4.956976
H	-1.892649	17.149605	4.968745
H	-6.256548	13.991587	4.882002
H	4.508121	19.371886	5.056139
H	11.611608	5.601150	4.632242
H	14.588380	10.836826	4.733290
H	4.201536	16.609429	5.140086
H	16.864310	10.962822	4.705590
H	10.423801	7.344968	4.658317
H	18.995945	12.346069	4.774646
H	-1.354207	11.745349	5.066478
H	-3.500726	10.457888	4.981945
H	5.238920	14.761077	5.048466
H	8.125812	8.434701	4.746555
H	0.764066	11.698417	5.006785
H	12.074836	12.145001	4.692585
H	7.380486	13.504030	4.920237
H	9.585296	12.669364	4.776077
H	3.128848	10.148694	5.022911
H	5.611810	9.387377	4.898193
H	16.616287	3.483490	4.786286
H	-0.370207	21.712281	4.942345
H	12.177757	0.402037	4.845393
H	16.628016	0.912691	4.876865
N	-4.062960	11.306517	4.959187
N	3.092892	21.795691	4.974988
N	13.999121	10.022091	4.648693
N	17.282540	6.518908	4.744345
N	21.131073	9.615002	4.873484
N	12.610654	5.472718	4.679775
N	-1.553520	16.202189	5.043654
N	-5.561956	13.248655	4.912694

N	19.574832	11.509834	4.806508
N	3.223582	16.855639	5.100811
N	1.427536	12.450822	5.114206
N	8.528606	9.370181	4.743434
N	9.344440	11.680464	4.763852
N	6.902008	12.606621	4.916335
N	5.936364	10.352612	4.912694
N	0.646571	21.669773	4.956651
N	13.174561	0.605538	4.852156
N	15.610511	0.878948	4.863795
Ni	-5.882210	11.432853	4.888310
Ni	7.667738	10.980930	4.828617
Ni	1.784511	23.083088	4.914060
N	-6.154028	9.615002	4.873484
N	-7.710269	11.509834	4.806508
N	0.439312	24.289241	4.852156
N	2.875262	24.562651	4.863795
Ni	21.402891	11.432853	4.888310
Ni	14.519760	-0.600615	4.914060
C	14.823368	7.887092	1.429117
C	1.948816	20.742257	1.747218
C	13.411624	3.289782	1.504090
C	18.884917	10.114842	1.546617
C	-4.320035	13.404741	1.711715
C	-0.058627	19.270996	1.771408
C	11.956306	3.215793	1.492971
C	13.657466	7.102357	1.418127
C	1.368763	19.495383	1.783307
C	14.085281	4.527738	1.477560
C	-4.772307	15.863377	1.687655
C	3.817509	15.367076	1.838513
C	-0.233391	17.839115	1.803205
C	-3.402309	16.038547	1.732458
C	-1.312388	16.948024	1.790395
C	-5.247117	14.533646	1.672765
C	11.193041	4.400832	1.454411
C	13.335799	5.694331	1.442252
C	18.349737	8.802811	1.524313
C	12.311901	9.192429	1.388736
C	1.070745	17.256057	1.834872
C	11.889969	5.597598	1.430157
C	-1.152870	15.527853	1.806716
C	-2.464964	14.939692	1.768156
C	16.971922	8.675744	1.482437

C	13.514037	9.941584	1.393353
C	-2.931657	13.630657	1.763800
C	1.283055	15.881383	1.854119
C	0.150122	15.021082	1.841374
C	16.092375	9.818459	1.452135
C	12.414427	7.803908	1.395174
C	14.759200	9.317226	1.414551
C	17.995617	11.273205	1.518136
C	2.458776	15.069509	1.854444
C	16.604465	11.109827	1.467546
C	2.001428	13.699326	1.845081
C	4.733088	14.304769	1.800018
C	11.222238	10.118910	1.413381
C	2.871892	12.630993	1.821997
C	9.847956	9.957980	1.454931
C	11.797083	11.440070	1.423134
C	4.252491	12.929610	1.790330
C	9.046231	11.102430	1.516315
C	11.031375	12.595280	1.468847
C	9.627168	12.433977	1.522037
C	-0.919844	20.372137	1.726215
C	1.081716	21.844067	1.697214
C	-0.361844	21.667070	1.687916
H	-3.068905	18.147507	1.696239
H	10.505604	1.817077	1.525614
H	-5.468719	16.704147	1.659890
H	3.029290	20.882530	1.754826
H	15.174982	4.537778	1.488354
H	-7.229296	14.856269	1.595905
H	19.013840	7.939248	1.545511
H	16.502155	6.589886	1.533742
H	20.866054	9.747905	1.620485
H	10.101384	4.367605	1.446348
H	2.997898	18.138342	1.870245
H	0.021126	12.902666	1.769197
H	10.407970	7.130063	1.362401
H	-2.254804	12.776670	1.796897
H	4.185705	16.394524	1.844170
H	-4.386439	11.393422	1.727841
H	15.963712	11.994143	1.436009
H	18.110232	13.281603	1.520542
H	9.370586	8.978414	1.454021
H	6.483694	15.304790	1.755932
H	13.845440	12.041075	1.495702

H	2.534670	11.594491	1.809902
H	7.229019	10.235461	1.582250
H	5.024209	11.070128	1.726410
H	11.480657	13.590798	1.479576
H	8.997695	14.352115	1.604294
H	-2.006781	20.256001	1.716201
H	14.979712	2.027211	1.560142
H	2.431749	23.337454	1.657094
H	-2.018511	22.826801	1.625622
N	18.672465	12.432975	1.543300
N	11.516614	1.943801	1.527499
N	0.610384	13.717401	1.853794
N	-2.673035	17.220584	1.758142
N	-6.521568	14.124490	1.629003
N	1.998852	18.266773	1.822712
N	16.163026	7.537302	1.458833
N	20.171461	10.490836	1.589793
N	-4.965326	12.229657	1.695979
N	11.385923	6.883852	1.401676
N	13.181969	11.288669	1.388281
N	6.080899	14.369311	1.759053
N	5.265066	12.059027	1.738635
N	7.707498	11.132870	1.586152
N	8.673141	13.386880	1.589793
N	13.962934	2.069719	1.545836
N	1.434944	23.133953	1.650331
N	-1.001005	22.860544	1.638692
Ni	20.491716	12.306638	1.614177
Ni	6.941767	12.758562	1.673870
Ni	12.824995	0.656404	1.588428
N	20.763533	14.124490	1.629003
N	22.319775	12.229657	1.695979
N	14.170194	-0.549750	1.650331
N	11.734244	-0.823160	1.638692
Ni	-6.793385	12.306638	1.614177
Ni	0.089746	24.340107	1.588428

Supplementary References:

1. The CP2K developers group, http://www.cp2k.org/, 2012.
2. VandeVondele, J., Krack, M., Mohamed, F., Parrinello, M., Chassaing, T., and Hutter, J. QUICKSTEP: Fast and accurate density functional calculations using a mixed Gaussian and plane waves approach. *Comput. Phys. Commun.* **2005**, *167*, 103−128.
3. Perdew, JP; Ruzsinszky, A; Csonka, GI; Vydrov, OA; Scuseria, GE; Constantin, LA; Zhou, X; Burke, K. Restoring the Density-Gradient Expansion for Exchange in Solids and Surfaces. *Phys. Rev. Lett.* **2008**, *100*, 136406-136409.
4. VandeVondele, J., and Hutter, J. Gaussian basis sets for accurate calculations on molecular systems in gas and condensed phases. *J. Chem. Phys.* **2007**, *127*, 114105.
5. Lippert, G., Hutter, J., and Parrinello, M. A hybrid Gaussian and plane wave density functional scheme. *Mol. Phys.* **1997**, *92*, 477−487.
6. Lippert, G., Hutter, J., and Parrinello, M. The Gaussian and augmented-plane-wave density functional method for ab initio molecular dynamics simulations. *Theor. Chem. Acc.* **1999**, *103*, 124−140.
7. Goedecker, S., Teter, M., and Hutter, J. Separable dualspace Gaussian pseudopotentials. *Phys. Rev. B* **1996**, *54*, 1703−1710.
8. Hartwigsen, C., Goedecker, S., and Hutter, J. Relativistic separable dual-space Gaussian pseudopotentials from H to Rn. *Phys. Rev. B* **1998**, *58*, 3641−3662.
9. Grimme, S., Ehrlich, S. and Goerigk, L. Effect of the damping function in dispersion corrected density functional theory. *J. Comput. Chem.* **2011**, *32*, 1456−1465.
10. S. L. Dudarev, G. A. Botton, S. Y. Savrasov, C. J. Humphreys, and A. P. Sutton. Electron-energy-loss spectra and the structural stability of nickel oxide: An LSDA+U study. *Phys. Rev. B* **1998**, *57*, 1505.
11. Zhang, L. C., Zhang, L., Qin, G., Zheng, Q. R., Hu, M., Yan, Q. B., and Su, G. (2019). Two-dimensional magnetic metal–organic frameworks with the Shastry-Sutherland lattice. *Chem. Sci.* **2016**, *10*, 10381.
12. Frisch, M. J.; Trucks, G. W.; Schlegel, H. B.; Scuseria, G. E.; Robb, M. A.; Cheeseman, J. R.; Scalmani, G.; Barone, V.; Petersson, G. A.; Nakatsuji, H.; Li, X.; Caricato, M.; Marenich, A. V.; Bloino, J.; Janesko, B. G.; Gomperts, R.; Mennucci, B.; Hratchian, H. P.; Ortiz, J. V.; Izmaylov, A. F.; Sonnenberg, J. L.; Williams; Ding, F.; Lipparini, F.; Egidi, F.; Goings, J.; Peng, B.; Petrone, A.; Henderson, T.; Ranasinghe, D.; Zakrzewski, V. G.; Gao, J.; Rega, N.; Zheng, G.; Liang, W.; Hada, M.; Ehara, M.; Toyota, K.; Fukuda, R.; Hasegawa, J.; Ishida, M.; Nakajima, T.; Honda, Y.; Kitao, O.; Nakai, H.; Vreven, T.; Throssell, K.; Montgomery Jr., J. A.; Peralta, J. E.; Ogliaro, F.; Bearpark, M. J.; Heyd, J. J.; Brothers, E. N.; Kudin, K. N.; Staroverov, V. N.; Keith, T. A.; Kobayashi, R.; Normand, J.; Raghavachari, K.; Rendell, A. P.; Burant, J. C.; Iyengar, S. S.; Tomasi, J.; Cossi, M.; Millam, J. M.; Klene, M.; Adamo, C.; Cammi, R.; Ochterski, J. W.; Martin, R. L.; Morokuma, K.; Farkas, O.; Foresman, J. B.; Fox, D. J. Gaussian 16 Rev. C.01, Wallingford, CT, 2016.
13. Grimme, S.; Ehrlich, S.; Goerigk, L. Effect of the damping function in dispersion corrected density functional theory. *J. Comput. Chem.* **2011**, *32*, 1456-1465.
14. Lu, Y.; Hu, Z.; Petkov, P.; Fu, S.; Qi, H.; Huang, C.; Liu, Y.; Huang, X.; Wang, M.; Zhang, P.; Kaiser, U.; Bonn, M.; Wang, H. I.; Samori, P.; Coronado, E.; Dong, R.; Feng, X., Tunable

Charge Transport and Spin Dynamics in Two-Dimensional Conjugated Metal-Organic Frameworks. *J. Am. Chem. Soc.* **2024**, *146*, 2574-2582.
